# Central and peripheral myeloid-derived suppressor cell-like cells are closely related to the clinical severity of multiple sclerosis

**DOI:** 10.1007/s00401-023-02593-x

**Published:** 2023-05-27

**Authors:** María Cristina Ortega, Rafael Lebrón-Galán, Isabel Machín-Díaz, Michelle Naughton, Inmaculada Pérez-Molina, Jennifer García-Arocha, Jose Manuel Garcia-Dominguez, Haydee Goicoechea-Briceño, Virginia Vila-del Sol, Víctor Quintanero-Casero, Rosa García-Montero, Victoria Galán, Leticia Calahorra, Celia Camacho-Toledano, María Luisa Martínez-Ginés, Denise C. Fitzgerald, Diego Clemente

**Affiliations:** 1 Grupo de Neuroinmuno-Reparación, Hospital Nacional de Parapléjicos, SESCAM, Finca “La Peraleda” s/n, 45071 Toledo, Spain; 2 Centro de Investigación Biomédica en Red de Enfermedades Neurodegenerativas (CIBERNED), Carlos III Health Institute, c/Monforte de Lemos, 3-5, 28029 Madrid, Spain; 3 Wellcome-Wolfson Institute for Experimental Medicine, School of Medicine, Dentistry and Biomedical Science, Queen’s University Belfast, 97 Lisburn Rd, Belfast, BT9 7BL Northern Ireland UK; 4 Departamento de Neurología, Hospital Universitario de Toledo, Av. del Río Guadiana, 45007 Toledo, Spain; 5 Departamento de Neurología, Hospital General Universitario Gregorio Marañón, Calle del Dr. Esquerdo 46, 28007 Madrid, Spain; 6 Servicio de Citometría de Flujo, Hospital Nacional de Parapléjicos, SESCAM, Finca “La Peraleda” s/n, 45071 Toledo, Spain

**Keywords:** Biomarker, EAE, Histopathology, Regulatory cells, PBMCs, Myelin

## Abstract

Multiple sclerosis (MS) is a highly heterogeneous demyelinating disease of the central nervous system (CNS) that needs for reliable biomarkers to foresee disease severity. Recently, myeloid-derived suppressor cells (MDSCs) have emerged as an immune cell population with an important role in MS. The monocytic-MDSCs (M-MDSCs) share the phenotype with Ly-6C^hi^-cells in the MS animal model, experimental autoimmune encephalomyelitis (EAE), and have been retrospectively related to the severity of the clinical course in the EAE. However, no data are available about the presence of M-MDSCs in the CNS of MS patients or its relation with the future disease aggressiveness. In this work, we show for the first time cells exhibiting all the bona-fide phenotypical markers of M-MDSCs associated with MS lesions, whose abundance in these areas appears to be directly correlated with longer disease duration in primary progressive MS patients. Moreover, we show that blood immunosuppressive Ly-6C^hi^-cells are strongly related to the future severity of EAE disease course. We found that a higher abundance of Ly-6C^hi^-cells at the onset of the EAE clinical course is associated with a milder disease course and less tissue damage. In parallel, we determined that the abundance of M-MDSCs in blood samples from untreated MS patients at their first relapse is inversely correlated with the Expanded Disability Status Scale (EDSS) at baseline and after a 1-year follow-up. In summary, our data point to M-MDSC load as a factor to be considered for future studies focused on the prediction of disease severity in EAE and MS.

**Supplementary Information:**

The online version contains supplementary material available at 10.1007/s00401-023-02593-x.

## Introduction

Multiple sclerosis (MS) is an incurable immune-mediated demyelinating disease of the CNS. Its clinical course severity highly varies among patients [41]. Around 85% of patients present relapsing–remitting MS (RRMS), characterized by episodes of neurologic dysfunction (relapses) followed by periods of remission [36]. This has led to the idea that the control of immune activation plays a relevant role in MS [5]. In this regard, disease-modifying treatments (DMTs) have highlighted the involvement of immunoregulatory mechanisms in MS recovery [42].

The variability in clinical course severity poses a challenge to neurologists in choosing among DMTs. Although MRI allows us to obtain diagnostic information [7, 41], no single biomarker can predict disease severity. More intense inflammation is associated with a higher degree of cortical demyelination and a more severe clinical course [23]. Notably, patients with severe clinical courses show higher lesion load and more active lesions [22]. However, little is known about the relationship between regulatory myeloid cells and the heterogeneous severity of the MS [9, 16].

Myeloid-derived suppressor cells (MDSCs) are involved in the regulation of the immune response [43]. MDSCs comprise two subsets: polymorphonuclear-MDSCs (PMN-MDSCs) and monocytic-MDSCs (M-MDSCs), characterized by the differential expression of Gr-1 epitopes in murine cells and CD14/CD15 in humans [43]. The immunoregulatory role of MDSCs has been demonstrated in the experimental autoimmune encephalomyelitis (EAE) MS model [31, 48], where M-MDSCs (CD11b^+^Ly-6C^hi^Ly-6G^−/low^, also called Ly-6C^hi^ cells) have stronger immunosuppressive activity than PMN-MDSCs (CD11b^+^Ly-6C^int^Ly-6G^hi^) [6, 48]. Importantly, there is a correlation between splenic Ly-6C^hi^-cells and the previous severity of the clinical course in EAE [27, 31]. The pharmacological improvement of M-MDSC number attenuates clinical signs in MS animal models [26, 29]. Thus, Ly-6C^hi^-cells/M-MDSCs appear as relevant factors to make individualized associations with the severity of MS clinical course.

In human diseases, M-MDSCs are classified as CD11b^+^CD33^+^HLA-DR^−/low^CD14^+^CD15^−^cells [17, 43]. Currently, M-MDSCs have been studied in the peripheral blood of MS patients with controversial results: some authors described an irrelevant variation of M-MDSCs during MS clinical course [17], while others observed its increase during relapses [12, 13]. Notably, M-MDSCs show stronger immunosuppressive function during relapses and the proportion of circulating M-MDSCs in MS patients is tenfold higher than PMN-MDSCs [12, 17]. Hence, M-MDSCs appear to be the most relevant subset to enhance immune-regulation during MS relapse. However, there are no data about the relationship between M-MDSCs and the MS clinical severity or about their presence in the CNS of MS patients, important prior steps before considering them as a decision-making tool for neurologists.

In this work, we describe for the first time that infiltrated myeloid cells with the complete M-MDSC phenotype are differentially present in MS tissue, being correlated with longer disease durations in primary progressive MS (PPMS). Ly-6C^hi^-cells in peripheral blood at the onset of EAE showed immunosuppressive activity and are inversely correlated with the clinical course severity and the histopathological damage. Furthermore, our data from untreated MS patients show an inverse correlation between the abundance of M-MDSCs at an early time point of their clinical course and the EDSS at baseline and 1 year later. Hence, our data suggest that peripheral M-MDSCs are important factors to be considered for future therapeutic strategies related to differential clinical severity as well as for further studies focused on biomarker discovery to help predict the severity of the clinical course in MS.

## Materials and methods

### Human tissue

Snap-frozen tissue (MS) was supplied by UK MS Tissue Bank and formalin-fixed paraffin-embedded (FFPE) MS tissue (MSD) was provided by Prof. Denise Fitzgerald from the Dame Ingrid V. Allen tissue collection (Belfast, UK). *Post-mortem* MS cortical snap-frozen tissue (*n* = 20 secondary progressive MS-SPMS; *n* = 13 PPMS) and six matched controls (Ct) were analyzed (Table1). Clinical course of MS patients were classified as long or short according to the median of the disease duration [SPMS: 16 years (y), inter quartile range-IQR (13.5–34.5); PPMS: 17 y (11–28.5)].

**Table 1:** Demographic and clinical characteristics of MS patients and control subjects

Patient	Clinical course	MS	Age/sex	TP (h)	DD (y)	Type of lesion		
						AL	AIL	IL
MS122	Short (DD ≤ 16 y)	SP	44/M	16	16			1
MS356		SP	45/F	10	16	1		
MS408		SP	39/M	21	10	2	2	
MS422		SP	58/M	25	13	1		1
MS371		SP	40/M	27	14	1		
MS114		SP	52/F	12	15			1
MS163		SP	45/F	28	6	1		
MS 154		SP	34/F	12	12	1	1	1
MS160		SP	44/F	18	16	4	1	1
MSD15		SP	38/M	nd	14	1		
MSD70		SP	49/M	nd	13		2	
MS318	Long (DD > 16 y)	SP	59/F	13	34	3	2	
MS187		SP	57/F	13	27		1	1
MS404		SP	55/F	17	34	1	2	1
MS466		SP	65/F	25	36	1	1	
MS406		SP	62/M	23	47	3		
MS470		SP	64/F	nd	35	2	2	2
MS055		SP	47/F	15	32	2	1	3
MS330		SP	59/F	21	39			1
MSD58		SP	82/M	nd	62			1
MS383	Short (DD ≤ 17 y)	PP	42/M	17	17	3	1	
MS325		PP	51/M	13	2	1		
MS083		PP	54/M	13	13		1	2
MS094		PP	42/F	11	6	3	3	1
MS473		PP	39/F	9	13	4	2	
MS216		PP	58/F	9	12	1		
MS500		PP	50/M	32	8	5		
MSD276		PP	40/F	nd	17		1	
MS057	Long (DD > 17 y)	PP	77/F	9	47	1	1	1
MS168		PP	88/F	22	30	2		2
MS492		PP	66/F	15	31			1
MS398		PP	57/F	28	28			1
MS363		PP	42/M	20	27	2	3	1
CO22		Ct	69/F	33				
CO72		Ct	72/M	26				
CO73		Ct	71/M	29				
CO74		Ct	84/F	22				
PDCO23		Ct	78/F	23				
PDCO28		Ct	84/F	11				

CNS samples from MS patients provided by the UK MS Tissue Bank (London, UK, identified as MS) and Dame Ingrid V. Allen tissue collection (from Belfast, UK, identified as MSD)

AL active lesion, AIL mixed active/inactive lesion, Ct control, DD disease duration, F female, h hours, IL inactive lesion, M male, nd not determined, PP primary progressive, SP secondary progressive, TP time *post-mortem,* y years

### Immunohistochemistry and eriochrome cyanine for myelin staining

Cryosections (10 µm, Leica) from snap-frozen tissue were fixed in 4% paraformaldehyde (PFA: Sigma-Aldrich) for 1 h. Immunohistochemistry (IHC) or immunofluorescence (IF) staining was performed as described previously [4] by incubating with the following primary antibodies: anti-CD11b (1:100; Abcam, ab133357); anti-CD3 (1:200; Agilent, A0454); anti-GFAP (1:500; Merck, MAB3402, GA5 clone); anti-TMEM119 (1:50; Sigma-Aldrich, HPA0518870); anti-CD33 (1:50; Merck, 133 M-14, PWS44 clone), anti-CD14 (1:25; R&D, BAF383), anti-CD84 (1:200; Invitrogen, PA5-64444), anti-CD15 (1:25; Agilent, ISO62, carb-3 clone), and anti-human leukocyte antigen (HLA)-DR (1:200 for IHC, 1:100 for IF; Agilent, M0746, TAL.1B5 clone). Antigen retrieval with citrate buffer 0.1 M pH 6.0 at 90ºC for 10 min was performed for CD33 labeling. Detection of TMEM119 and CD33 was visualized using TSA signal amplification system (Tyramide SuperBoost™ kit, Invitrogen for TMEM119 and TSA Plus biotin kit, AkoyaBio for CD33). Appropriate fluorescent-tagged (1:1000, Invitrogen) or biotinylated (1:200; Vector Labs) secondary antibodies were used. The IHC reaction was developed using the Vectastain Elite ABC reagent (Vector Labs) and the peroxidase reaction product was visualized with 0.05% 3, 3´-diaminobenzidine (DAB, Sigma-Aldrich) and 0.003% H_2_O_2_ in 0.1 M Tris–HCl, pH 7.6. The reaction was monitored under the microscope and terminated by rinsing the slides with PB. Fluorescent Hoechst 33342 staining (10 µg/ml: Sigma-Aldrich) was used for cell nuclei detection. Negative stain controls were included and the absence of the appropriate antibodies yielded no signal.

To visualize myelin after HLA-DR immunostaining in tissue from snap-frozen brain blocks, eriochrome cyanine (EC) staining was carried out as described. The sections were air-dried overnight at RT and for 2 h at 37 °C in a slide warmer. The sections were then placed in fresh acetone for 5 min and air-dried for 30 min, before they were stained in 0.5% EC for 1 h and differentiated in 5% iron alum and borax-ferricyanide for 10 and 5 min, respectively (briefly rinsing the sections in tap water between each step). After washing with abundant water, correct differentiation was assessed under the microscope whereby the myelinated areas were stained blue and the demyelinated areas appeared white-yellowish. The stained sections were dehydrated and mounted for preservation at RT.

Triple chromogenic immunohistochemistry on FFPE sections (from MSD tissue blocks) was performed with a triple stain IHC kit (DAB, AP/Red & HRP/Green) from Abcam (ab183286) which allowed red–green colocalisation using a modified protocol. FFPE sections were dewaxed in clearene and rehydrated through a graded series of alcohols. Heat-induced epitope retrieval was conducted in a steamer for 1 h, while slides were incubated in sodium citrate buffer (pH 6.0). Endogenous peroxidases were blocked with 0.3% H_2_O_2_ in methanol before blocking with 5% normal goat serum in Tris-buffered saline (TBS, pH 7.4). Slides were then incubated with anti-CD15 (1:50; Agilent, ISO62, carb-3 clone) in blocking solution overnight. Slides were washed in TBS before EnVision anti-mouse HRP secondary antibody (Agilent) was applied for 30 min at RT. Antibody binding was visualized with DAB (Immpact DAB; Vector) as chromogen. To prevent cross-reactivity, slides were heated to 80 °C in supplied antibody blocker solution and then incubated with Blocker A and B according to the manufacturer’s protocol. Slides were washed in TBS before incubation with anti-HLA-DR antibody (1:200; Agilent, M0746, TAL.1B5 clone) and anti-CD14 (1:50; R&D, BAF383) in blocking solution overnight at 4 °C. After washing, tissue sections were incubated with kit-supplied anti-mouse AP secondary antibody for 30 min at RT. Followed further washing, sections were incubated with an ABC peroxidase-linked reporter system (Vector Laboratories) for 30 min at RT. Detection of HLA-DR was visualized with Permanent Red chromogen and counterstained with Gill’s haematoxylin No. 2 (GHS232; Sigma) diluted 1:10. Slides were briefly washed and detection of CD14 was visualized by applying Emerald Green chromogen for 5 min at RT. Slides were rapidly cleared and mounted with supplied mounting medium according to the manufacturer’s instructions. Negative and single stain controls were included, and in all instances, the absence of the relevant antibodies yielded no signal.

### Classification of MS lesions

MS lesions were classified according to demyelination and cellular distribution as described [4, 18, 22]. Briefly, active lesions (AL) are demyelinated areas loaded by dense infiltration of macrophage/microglial cells. T cells were observed both perivascularly and dispersed in the lesion and astrogliosis were confirmed by GFAP upregulation. Mixed active/inactive lesions (AIL) are demyelinated areas with a hypocellular lesion center (cAIL) surrounded by a lesion rim (rAIL) enriched with macrophage/microglial cells. Moderate T-cell infiltration was observed in the center of AILs and the presence of hypertrophied astrocytes was also found. Inactive lesions (IL) contained very few macrophage/microglial cells and T cells within the demyelinating lesion. Gliotic scar formed by astrocytes was found in the center of the ILs.

### Cell counting in human tissue

The IHC staining for HLA-DR, CD14, and CD15 was used for the quantification of M-MDSCs in serial sections of each case and lesion. To measure HLA-DR fluorescence intensity, photomicrographs of the MS lesions were acquired as a mosaic of 20X magnification images captured on a confocal microscope equipped with a resonant scanning system (SP5: Leica), quantifying the fluorescence intensity of the cells using the ImageJ software. A low level of HLA-DR fluorescence was established in a blinded manner by measuring HLA-DR staining in 100 CD14^+^ CD15^−^ cells in each patient, among which there were 25 cells with no HLA-DR immunostaining, 25 cells with very faint staining (HLA-DR^low^), and 50 cells with mild-to-strong immunostaining (HLA-DR^int/high^: Suppl. Fig. 1a). When the maximum fluorescence intensity was quantified in each HLA-DR cell from each patient, a receiver-operating characteristic (ROC) curve analysis was performed to find the optimal cut-off to accurately classify HLA-DR^low^ cells avoiding false-positive cells, i.e., cells that were classified as HLA-DR^low^ by the blinded observer but with a fluorescence intensity above the cut-off value. Once this threshold was calculated for each patient, only those cells with HLA-DR fluorescence intensity below the cut-off value were considered to quantify the M-MDSC density (Suppl. Fig. 1b). Due to the variation of HLA-DR staining between patients, quantification and cut-off value determination were carried out on an individualized basis for each patient. The density of M-MDSCs was obtained by manually counting of HLA-DR^−/low^ CD14^+^ CD15^−^ cells within the MS lesions, using 5–15 fields of the area of interest at a magnification of 20X, depending on the size of the lesions (SP5: Leica).

Color deconvolution was performed to quantify M-MDSCs (HLA-DR^−/low^ CD14^+^ CD15^−^ cells) in the MS lesions from FFPE tissue from the Dame Ingrid V. Allen tissue collection (Belfast, UK). The color deconvolution plugin for Fiji implements stain separation with Ruifrok and Johnston’s method previously described [38]. The plugin allows us to transform each single staining from the multiple immunolabelling into a separate channel to analyze the pictures and quantified the density of MDSCs in different MS lesions as described above (Suppl. Fig. 1).

### Induction of EAE

EAE was induced 6-week-old C57/BL6 mice from both sexes (Janvier Labs) by immunization with 200 µg of Myelin Oligodendrocyte Glycoprotein (MOG_35-55_) peptide (GenScript) as previously described [31]. EAE was scored clinically on a daily basis in a double-blind manner [29, 31]. All animal manipulations were approved by the institutional ethical committees (*Comité Ético de Experimentación Animal, Hospital Nacional de Paraplejicos-HNP*), and all the experiments were performed in compliance with the European guidelines for animal research (European Communities Council Directive 2010/63/EU, 90/219/EEC, Regulation No. 1946/2003).

The clinical parameters analyzed were defined as: (i) the severity index (SI), quantified as the ratio between the maximal clinical score at peak and the disease duration (i.e., days elapsed from the onset to the peak of the disease [27]; (ii) the accumulated clinical score was considered as the sum of the individual clinical scores from the day of onset or the peak of the disease, until the end of the clinical evaluation; (iii) the percentage of recovery was determined as the following percentage: (the maximal clinical score at peak − the residual score in the plateau phase) × 100/maximal clinical score at peak; and (iv) the recovery index, as the absolute score recovered from the peak to the plateau phase/days elapsed from the peak to the end of the remission phase.

### Flow cytometry of peripheral blood cells from EAE mice

Blood was collected in 2% EDTA tubes from the orbital vein of 16 isoflurane-anesthetized mice at disease onset (clinical score ≥ 0.5) and at the peak of the EAE. After blocking Fc receptors, cells were labeled with the following antibodies: anti-Ly-6C FITC (10 µg/mL, AL-21 clone), anti-Ly-6G PE (4 µg/ml, 1A8 clone), and anti-CD11b PerCP-Cy5.5 (4 µg/ml, M1/70 clone) all from BD Biosciences; anti-MHC-II PE-Cy7 (4 µg/ml, M5/114.15.2 clone), anti-CD11c APC (4 µg/ml, N418 clone), and anti-F4/80 eFluor450 (4 µg/ml, BM8 clone) from eBioscience. Analysis was performed in an FACS Canto II cytometer (BD Biosciences) and data analysis was assessed using FlowJo 10.6.2 software (FlowJo, LLC-BD Biosciences).

### Immunosuppression assay with circulating Ly-6C^hi^ cells of EAE mice

Splenocytes were obtained from MOG-immunized female C57BL/6 mice at the onset of clinical score (0.5–1.5, Suppl. Fig. 2a), as described previously [31, 32]. Splenocytes were exposed to 5 µM Tag-it Violet™ Proliferation and Cell Tracking Dye (Biolegend) diluted in PBS supplemented with 0.1% BSA at 37 °C for 20 min. After washing, 2 × 10^5^ splenocytes were plated in EX-VIVO (Lonza) culture medium supplemented with 1% HEPES and 6 µM 2-ME (Sigma) in U-bottom 96-well plates and stimulated for 24 h with 5 µg/mL MOG. Then, Ly-6C^hi^-cells (CD11b^+^ Ly-6C^hi^ Ly-6G^−/low^ -cells) were sorted by FACS Aria Ilu from the whole blood of different EAE mice at disease onset (clinical score 0.5–1.5) and 5 × 10^4^ cells were plated in co-culture with the stimulated splenocytes (4:1, splenocytes:Ly-6C^hi^-cells). After 48 h, cells were harvested and stained with anti-CD11b PerCP-Cy5.5 (4 µg/ml, M1/70 clone), anti-CD3 APC (4 µg/ml; 145-2C11 clone), anti-CD4-PE (2 µg/ml; RM4-5 clone), and anti-CD8 FITC (5 µg/ml, 53–6.7 clone).

To analyze the T-cell proliferation without the suppressive effect from endogenous Ly-6C^hi^ cells, splenocytes from MOG-immunized EAE mice at the onset of the clinical score were depleted of Ly-6C^hi^ cells by cell sorting in a FACS Aria IIu (Suppl. Fig. 2b). After that, Ly-6C^hi^ depleted-splenocytes were labeled with Tag-it Violet™ Proliferation and Cell Tracking Dye and then MOG-stimulated as abovementioned. After 24 h, 5 × 10^4^ sorted Ly-6C^hi^ cells from the whole blood of other EAE mice at disease onset (clinical score ≥ 0.5) were added (4:1; depleted-splenocytes:Ly-6C^hi^ cells). After 72 h, cells were harvested and stained as previously described (see above).

Analysis was performed in an FACS Canto II cytometer (BD Biosciences) and data analysis was assessed using FlowJo 10.6.2 software (FlowJo, LLC-BD Biosciences).The proliferation index was calculated as the ratio of the percentage of stimulated divided cells with respect to control divided cells.

### Tissue extraction and histological analysis of EAE tissue

Ten female mice with EAE from a second cohort of animals were used for histological analysis. Peripheral blood was also collected from all the mice at the onset of the clinical signs and all the animals were sacrificed at the peak of the clinical course, when they were perfused transcardially with 4% PFA. The spinal cord of the mice was dissected out and post-fixed for 4 h at RT in the same fixative. After immersion in 30% (w/v) sucrose in PB for 12 h, coronal cryostat sections (20 μm thick: Leica) were thaw-mounted on Superfrost® Plus slides.

The same EC staining for myelin visualization was carried out as that used for the histopathology of human samples with the following modifications: the tissue was stained in 0.5% EC for 30 min, and differentiated in 5% iron alum and borax-ferricyanide for 10 and 5 min, respectively.

Axonal damage was analyzed by staining the non-phosphorylated form of the neurofilament protein (SMI-32) in spinal cord sections from mice with EAE at the peak. Immunohistochemistry was performed by incubating the sections overnight at 4 °C with SMI-32 antibody (1:200, Covance). After rinsing, the sections were then incubated for 1 h at RT with the corresponding fluorescent secondary antibody (1:1000; Invitrogen). The cell nuclei were then stained with Hoechst 33342 (10 µg/ml: Sigma-Aldrich), and the sections were mounted in Fluoromount-G (Southern Biotech).

### Image acquisition and analysis of murine tissue

In all cases, three sections from each thoracic spinal cord (separated by 420 μm) were selected from 10 EAE mice in the histological cohort. To measure demyelination, the EC stained spinal cord sections were analyzed on a stereological Olympus BX61 microscope, using a DP71 camera (Olympus) and VisionPharm software for anatomical mapping. Superimages were acquired at a magnification of 10× using the mosaic tool and analyzed with the Image J software, expressing the results as the percentage of white matter area with no signs of blue staining as well as the total demyelinated area.

To quantify axonal damage, mosaic images from the whole spinal cord of each animal were obtained on a DMI6000B microscope (Leica). The area of axonal damage relative to the total area or the infiltrated area was analyzed with an *ad-hoc* plugin designed by the Microscopy and Image Analysis Service at the *HNP*. Briefly, after selecting the appropriate area (the infiltrated area relative to the whole section or to the whole white matter area), a threshold for immunofluorescence was established and SMI-32 immunostaining was assessed, presenting the result as an area (μm^2^).

### MS patient cohort for M-MDSC blood analysis

All patients were diagnosed MS according to the revised 2017 McDonald criteria. The cohort included 47 untreated RRMS patients who had not received corticosteroids in the last 6 months and who experienced their first relapse up to 1 year before blood sampling (Suppl. Table 2). All MS patients were recruited at the Department of Neurology at Hospital Universitario Virgen de la Salud (Toledo, Spain) or at Hospital General Universitario Gregorio Marañón (Madrid, Spain). Peripheral blood samples were also obtained from matched healthy volunteers recruited in the *HNP*. The study was approved by the *Comité Ético de Investigación Clínica con Medicamentos* (#349) of the *Complejo Hospitalario de Toledo *and informed written consent was obtained from all participants in accordance with the Helsinki declaration.

### Flow cytometry of human peripheral blood mononuclear cells

Human peripheral blood mononuclear cells (PBMCs) were isolated by Ficoll density gradient centrifugation (GE-171440-02, Merck). Separated cells were subsequently collected from the interphase, washed with isolation buffer (2.23 g/l d-glucose, 2.2 g/L sodium citrate, 0.8 g/L citric acid, 0.5% BSA in PBS), and further centrifuged at 500 *g* for 10 min at RT. The cell pellet was resuspended in FBS, counted, and aliquoted 1:1 in FBS with 20% dimethyl sulfoxide (DMSO, Sigma-Aldrich). The samples were then stored in liquid nitrogen at − 160 °C until use.

Freshly thawed PBMCs (1 × 10^6^) were washed with RPMI and stained with Zombie NIR Dye (Biolegend) for living cell identification following the manufacturer’s instructions. Then, Fc receptors were blocked with beriglobin (50 μg/ml; CSL Behring) for 10 min at 4 °C. The antibody panel for MDSC analysis was made up of anti-CD15 FITC (1.25 µl/test, HI98 clone), anti-CD14 PerCP-Cy^TM^5.5 (0.5 µl/test, Mφ29 clone), anti-CD11b PE-Cy7 (0.5 µl/test, ICRF44 clone), anti-CD33 APC (1.25 µl/test, WM53 clone), and anti-HLA-DR BV421 (0.5 µl/test, G46-6 clone, all from BD Biosciences). PBMCs were fixed with 0.1% PFA and then were analyzed as described above for murine blood cells.

### Statistical analysis

Data were expressed as mean ± SEM and analyzed with SigmaPlot version 11.0 (Systat Software). To compare between different MS lesion types, a one-way ANOVA test was performed or its corresponding ANOVA on ranks, followed by the Tukey or Dunn *post hoc* tests, respectively. Student’s *t* test was used to perform two-by-two comparisons (Mann–Whitney *U* test for non-parametric data). Paired *t* test was used for comparison in the in vitro analysis of immunosuppressive activity. Shapiro–Wilk normality tests were performed on human MS tissue samples. Pearson or Spearman tests were used for correlations as appropriate. The ROC curve was quantified using the area under the curve (AUC). The minimal statistical significance was set at *p* < 0.05 and represented as: *, #*p* < 0.05; **, ##*p* < 0.01; ***, ###*p* < 0.001.

## Results

### M-MDSCs are present in the CNS of MS patients

**Fig. 1:**
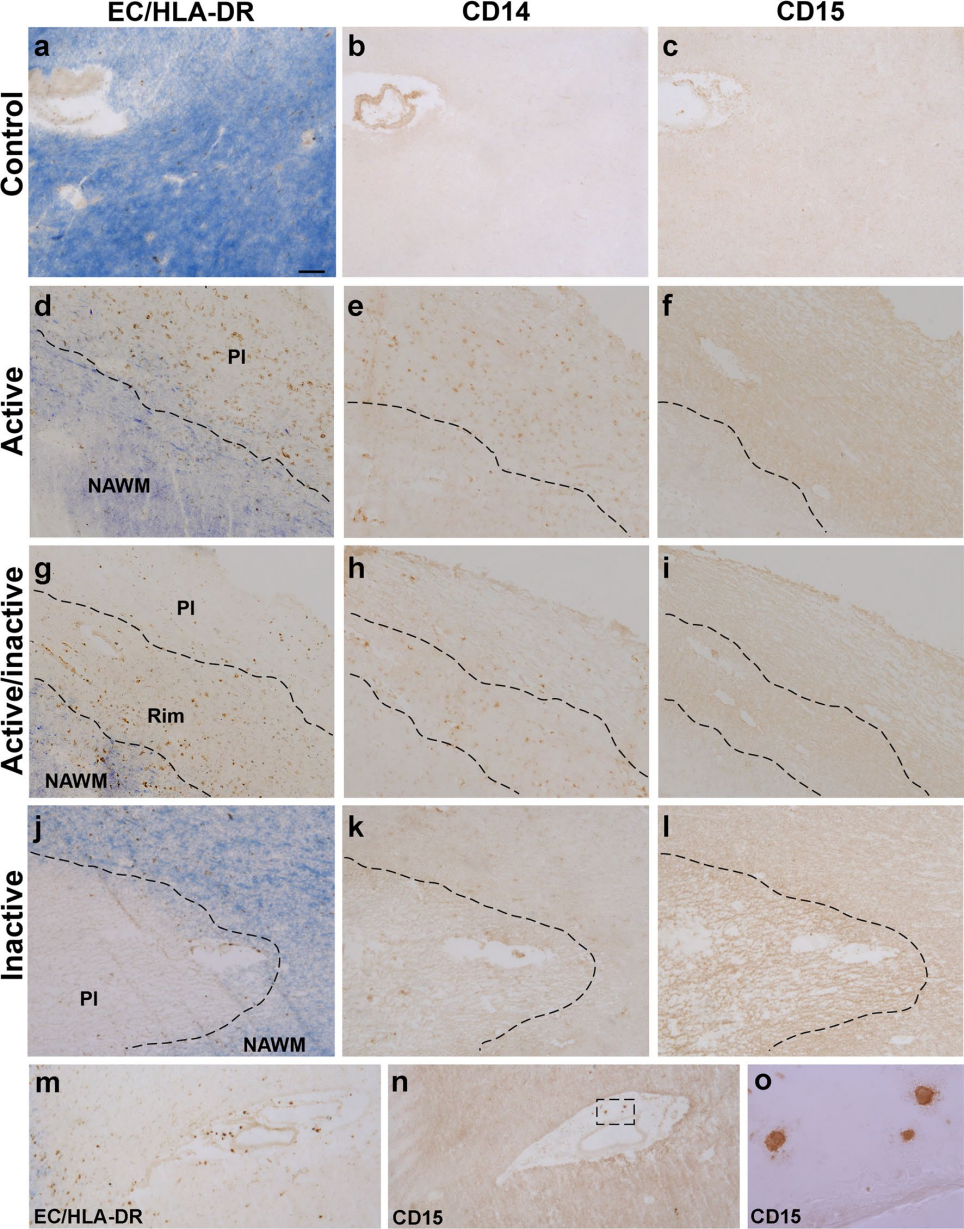
Expression pattern of CD14 and CD15 in MS lesions. **a**–**c** CD14 or CD15 staining was not detected in control human tissue. CD14^+^-cells were located both in the plaque of AL (**d**, **e**) and in the rim of AIL (**g, h**). By contrast, these cells were almost absent in the center or plaque of AIL (**g**, **h**) and IL (**j, k**). CD15 staining was not detected in any region of the MS lesions (**f**, **i**, **l**), although CD15^+^ granulocytes were clearly detected in the perivascular area of blood vessels (**m**–**o**). EC eriochrome cyanine, AL active lesions, AIL mixed active/inactive lesions, IL inactive lesions, Pl plaque, NAWM normal appearing white matter. *N* = 46 ALs, 26 AILs and 24 ILs from 33 MS patients Scale bar: **a**–**c**, **j**–**n** = 100 µm; **d**–**i** = 125 µm; **o** = 15 µm

We explore the presence of cells with M-MDSC phenotype in human tissue using their bona-fide peripheral blood markers, i.e., CD14^+^CD15^−^CD33^+^CD11b^+^HLA-DR^−/low^. Neither CD14 nor CD15 was observed in the white matter of control tissue (Fig. 1a–c). CD14^+^-cells were observed within AL and in the rAIL (Fig. 1d, e, g, h), being absent from cAIL and IL (Fig. 1g, h, j, k). No CD15 was detected in any MS lesion (Fig. 1f, i, l), being restricted to perivascular granulocytic-like cells (Fig. 1m–o).

**Fig. 2:**
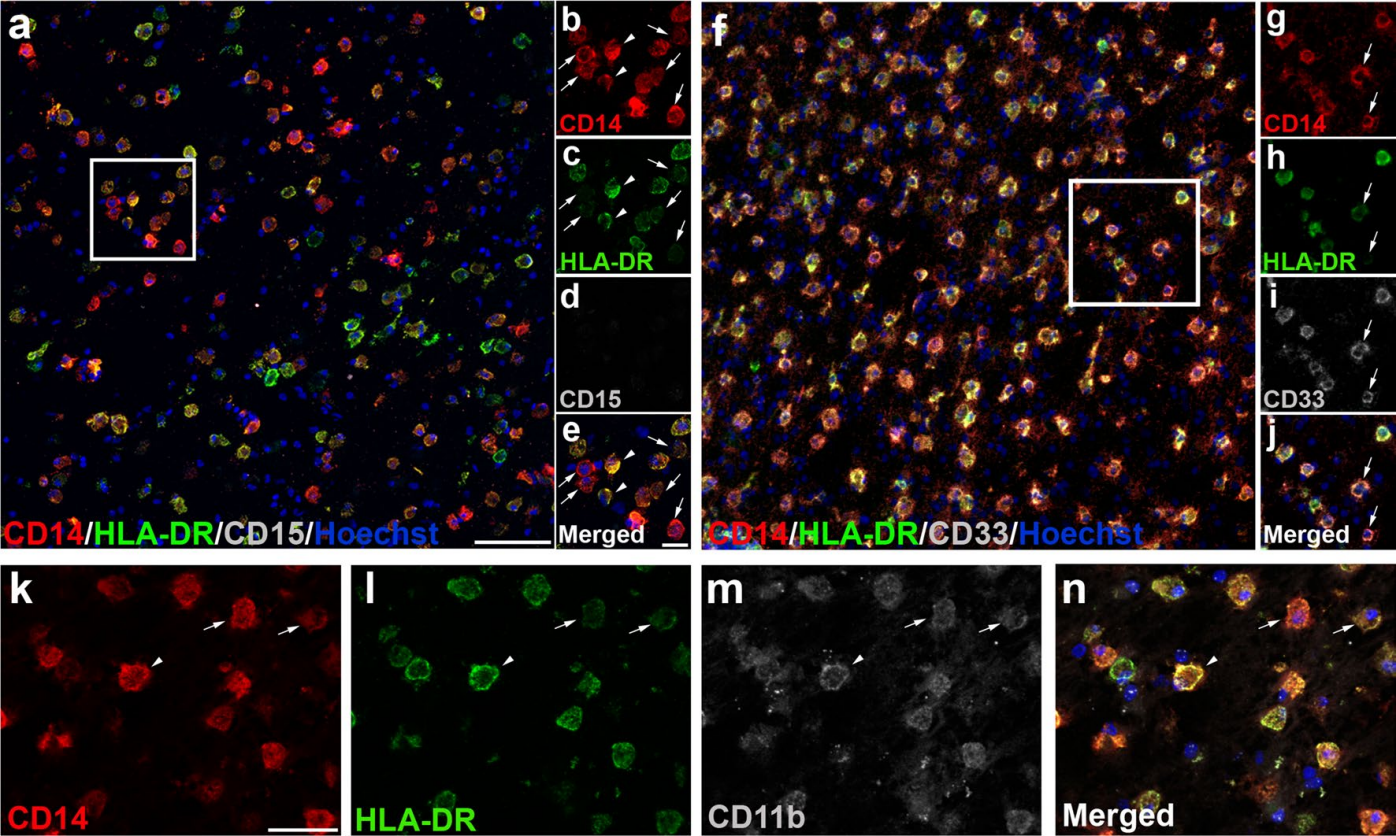
Immature myeloid monocytic cells are present within MS lesions. **a** Based on the intensity of HLA-DR, we identified different CD14^+^-cell subpopulations: CD14^+^HLA-DR^−/low^-cells were considered as M-MDSC-like cells (arrows in **b**–**e**) and CD14^+^HLA-DR^hi^-cells were identified as inflammatory macrophages (arrowheads in **b**–**e**). The lack of CD15 expression in CD14^+^-cells (**d**) corroborated the exclusive presence of M-MDSC-like cells. **f**–**g** CD33 as typical marker for immature myeloid cells was observed in CD14^+^HLA-DR^−/low^ (arrows in **g**–**j**). **k**–**n** CD14^+^-cell subpopulations were identified as myeloid cells by CD11b staining. Arrows point to M-MDSC-like cells (CD14^+^HLA-DR^−/low^-cells). Arrowheads indicate CD11b^+^ inflammatory macrophages (CD14^+^HLA-DR^hi^-cells). *N* = 46 ALs, 26 AILs, and 24 ILs from 33 MS patients. Scale bar: **a**, **f** = 100 µm; insets in **b**–**d** = 20 µm, insets in **g**–**j** = 30 µm; **k**–**n** = 50 µm

**Fig. 3:**
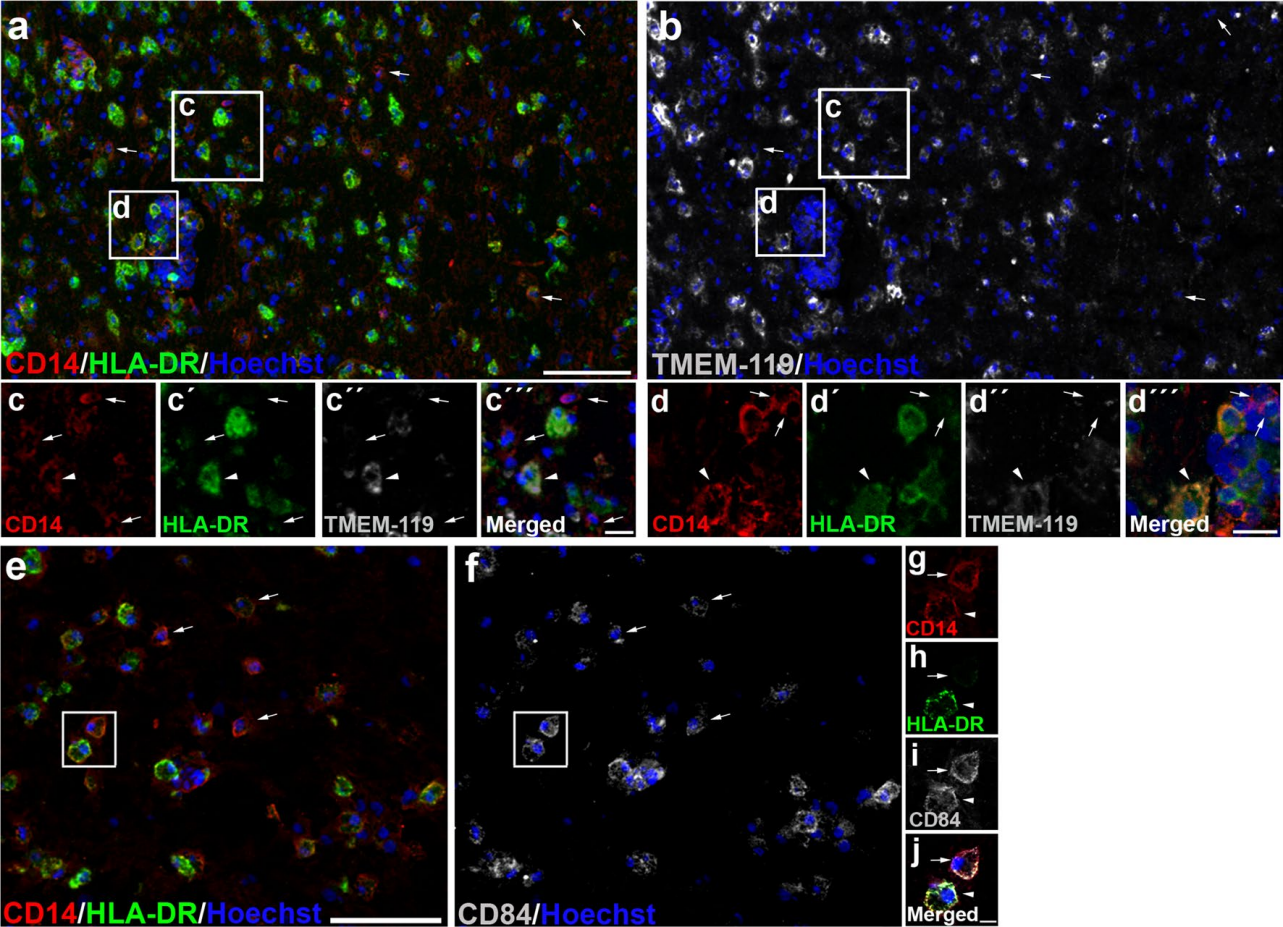
Infiltrated cells with the M-MDSC phenotype are observed within MS lesions. **a**, **b** The lack of TMEM119 immunoreactivity observed in amoeboid CD14^+^HLA-DR^−/low^ M-MDSC-like cells within ALs in both the brain parenchyma (arrows in **c**–**c**´´´) and in blood vessels-associated cells (arrows in **d**–**d**´´´) confirmed their peripheral origin. Arrowheads in **c**–**c**´´´and **d**–**d**´´´ point to inflammatory microglial-derived macrophages (CD14^+^HLA-DR^hi^TMEM119^+^) **e**–**j** The immunosuppressive marker CD84 was observed in some M-MDSC-like cells (arrows indicate CD14^+^HLA-DR^−/low^ CD84^+^-cells in **e**, **f** and **g**–**j**, while arrowhead points CD14^+^ HLA-DR^hi^-cells in **g**–**j**). *N* = 46 ALs, 26 AILs and 24 ILs from 33 MS patients. Scale bar: **a**, **b** = 100 µm; insets in **c**–**c**´´´ and **d**–**d**´´´ = 20 µm; **e**, **f** = 100 µm; insets in **g**–**j** = 10 µm

According to previous classifications of M-MDSCs based on HLA-DR expression [43], we established two cell subpopulations of CD14^+^-cells: CD14^+^HLA-DR^−/low^-cells were considered as M-MDSC-like cells and CD14^+^HLA-DR^hi^-cells were identified as pro-inflammatory macrophages (Fig. 2a–e). The lack of CD15 (Fig. 2d, e) and the presence of CD33 and CD11b (Fig. 2f–m) in all CD14^+^-cells corroborated their classification as monocytic myeloid cells. To be able to determine whether CD14^+^HLA-DR^−/low^-cells are derived from microglia or from inflammatory monocytes, we took advantage of TMEM119 labeling, as the only specific marker of microglia in human CNS [39]. It has been described that TMEM119 is absent in the core of AILs and in the center of ALs, but is still visible at the edge of ALs [46]. To avoid misinterpretations of the absence of TMEM119 labeling, we specifically checked the phenotype of CD14^+^HLA-DR^−/low^-cells in the rim of ALs, both in the parenchyma and in blood vessel-associated cells. Whereas TMEM119 was present in some CD14^+^HLA-DR^hi^ round-shaped cells (Fig. 3a, b, insets “c” and “d”), CD14^+^HLA-DR^−/low^-cells lacked TMEM119 immunoreactivity, not only in those cells within MS lesions (Fig. 3a, b, inset “c”), but also in blood vessel-associated cells (Fig. 3a, b, inset “d”), indicating their peripheral origin as monocyte-derived cells. Finally, we explored the presence in MS lesions of CD84, recently considered as a marker of the immunosuppressive activity of MDSCs [19, 20]. Accordingly, CD84 was present on a proportion of CD14^+^HLA-DR^−/low^ M-MDSCs, but also in some CD14^+^HLA-DR^hi^ pro-inflammatory myeloid cells (Fig. 3e–j), as was previously described [40]. We found no differences in the cellular phenotype of putative M-MDSCs between patients with PPMS and SPMS, or between the different areas of demyelinating lesions analyzed (not shown).

In summary, we provide the first description of a myeloid cell population expressing all the typical markers to be considered as infiltrated cells with an M-MDSC phenotype in the white matter lesions of MS patients.

### M-MDSCs are differentially associated with the severity of the clinical course in SPMS and PPMS patients

**Fig. 4:**
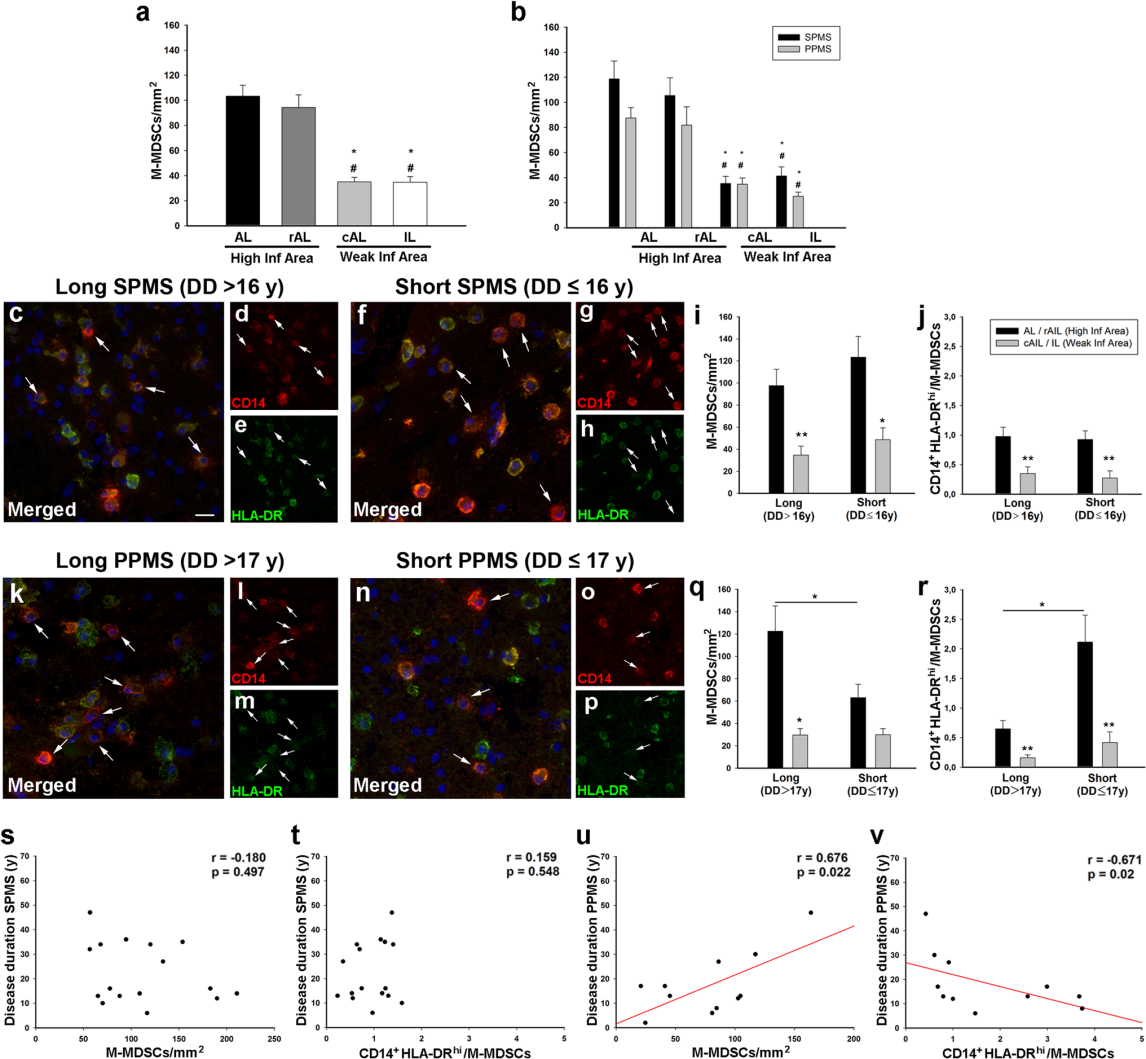
Distribution pattern of M-MDSCs in MS patients. **A**,**b** The density of M-MDSC-like cells was significantly higher in the AL and in the rAIL of MS patients. Representative images of M-MDSCs within high inflammatory areas of SPMS patients with long (**c**–**e**) or short (**f**–**h**) disease duration. Arrows indicate M-MDSCs. The density of M-MDSC (**i**) and the IIR (CD14^+^HLA-DR^hi^-cells/M-MDSCs, **j**) were higher in high inflammatory areas of SPMS independently of the disease duration. **k**–**p** Representative images of high inflammatory areas of PPMS patients with different disease duration. **q** Only PPMS patients with a long disease course had a significantly higher density of M-MDSCs in the high inflammatory areas. M-MDSC density was similar in high and weak inflammatory regions of PPMS patients with short disease duration. **r** The IIR significantly increased in higher inflammatory regions in those PPMS patients with short disease duration. There was no correlation between the density of M-MDSCs (**s**) or the IIR (**t**) and the clinical course duration in SPMS patients. The abundance of this regulatory cell population in high inflammatory areas (**u**) and the IIR (**v**) from PPMS patients were correlated with the disease duration. N = 46 ALs, 26 AILs and 24 ILs from 33 MS patients in **a**. *N* = 24 ALs, 14 AILs and 14 ILs from 20 SPMS patients; 22ALs, 12 AILs and 10 ILs from 13 PPMS patients in **b**. *N* = 11 SPMS and 8 PPMS with short DD, 9 SPMS and 5 PPMS with long DD in **i**, **j**, **q**, **r**. Scale bar: **c**, **f**, **k**, *n* = 20 µm; **d**, **e**, **g**, **h**, **l**, **m** and **o**, **p** = 40 µm. In **b**, * represents the difference with respect to AL and ^#^ with respect to the rAIL; High. Inf Area, high inflammatory area; Weak Inf Area, weak inflammatory area. DD disease duration

The study of the cells with the M-MDSC immunophenotype showed that (i) all cells expressed CD33 and CD11b marker, (ii) all of them lacked the TMEM119 marker, and (iii) CD84 was not a fully specific marker for M-MDSCs. For this reason, for the quantitative analysis in different regions of the white matter lesions, we used the commonly accepted markers in phenotyping of M-MDSCs, i.e., CD14^+^HLA-DR^−/low^CD15^−^, referring to them hereafter as M-MDSCs [44]. The quantification of M-MDSCs showed that their density was higher in demyelinating areas of AL or in the rAIL than in the cAIL or the IL (Fig. 4a). These regions are characterized by different inflammatory activity [18], so we gathered the data from the AL and the rAIL (high inflammatory area), and compared this with the results from the cAIL and IL (weak inflammatory area) showing that the distribution pattern showed no differences between SPMS and PPMS patients (Fig. 4b).

To explore the influence of disease severity on M-MDSC distribution, we compared their density in the CNS of patients with different clinical course duration according to the median value of the patients’ disease duration from SPMS or PPMS (Table 1). We did not observe differences between SPMS patients with long or short disease duration, being the highest M-MDSC density always present in high inflammatory areas (Fig. 4c–i). We also scrutinized whether the ratio between pro-inflammatory/immunosuppressive cells (IIR) would be affected by the disease course: similarly to M-MDSCs, the ratio of CD14^+^HLA-DR^hi^-cells/M-MDSCs was significantly higher in high inflammatory regions of SPMS, irrespectively of the disease duration (Fig. 4j). On the contrary, the density of M-MDSCs in high inflammatory areas was significantly lower in PPMS patients with short disease course (Fig. 4k–q). Remarkably, PPMS patients with short clinical course showed no differences in the M-MDSC density between high and weak inflammatory areas (Fig. 4q). Finally, IIR was dramatically increased in PPMS with short disease duration, suggesting a dampened immune-regulatory environment (Fig. 4r).

Correlation analysis showed that M-MDSCs density or IIR in high inflammatory areas of SPMS patients were independent of the disease duration (Fig. 4s, t). Both parameters were independent of the time *post-mortem* or age (not shown). Interestingly, the abundance of M-MDSCs in high inflammatory areas of PPMS patients was directly correlated with age (*r* = 0.613, *p* = 0.045) and disease duration (Fig. 4u), i.e., the lower the M-MDSC density in high inflammatory regions, the shorter the clinical course duration. In addition, the disease duration in PPMS patients showed an inverse correlation with the IIR in high inflammatory regions (Fig. 4v).

These findings demonstrate that the distribution of myeloid cells expressing all the typical markers for M-MDSCs was mainly found in high inflammatory areas of both SPMS and PPMS, being independent to clinical course duration in SPMS patients. However, a lower density of these regulatory cells in parallel with an exacerbated inflammatory context is directly correlated with shorter disease duration in PPMS patients.

### A higher abundance of blood Ly-6C^hi^-cells at onset is indicative of a milder EAE disease severity

**Fig. 5:**
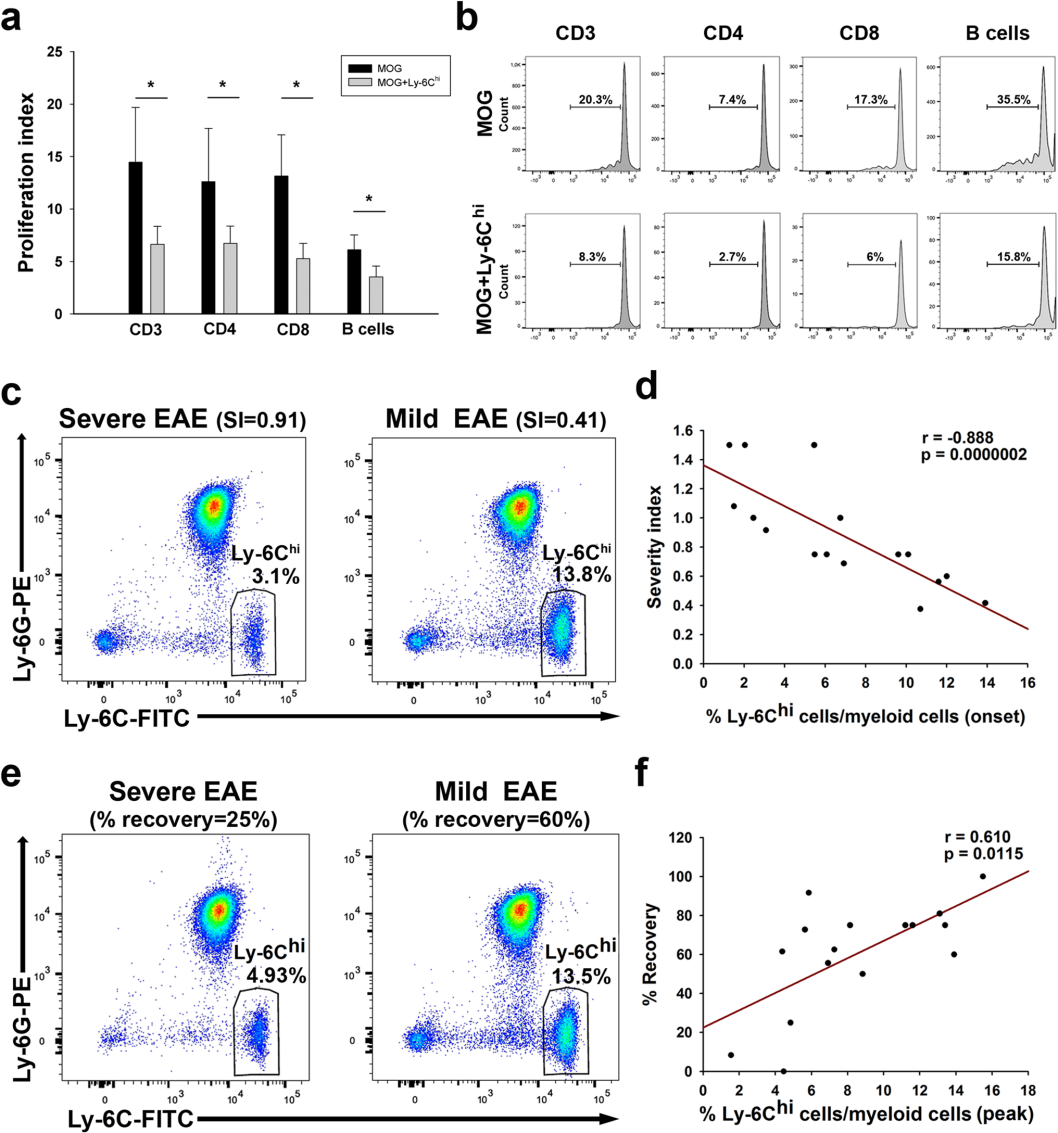
The proportion of immunosuppressive Ly-6C^hi^-cells in the peripheral blood is indicative of a less severe clinical evolution and a better recovery in the EAE model. **a** Circulating Ly-6C^hi^-cells from the EAE mice at the onset significantly decreased the proliferation of T and B cells within MOG-stimulated splenocytes after 48 h in co-culture. **b** Representative plots of the percentage of different populations of divided MOG-stimulated cells alone or in co-culture with Ly-6C^hi^-cells. **c** Representative flow cytometry plots of Ly-6C^hi^-cells from EAE mice with a severe (left panel) or mild (right panel) clinical course. **d** The abundance of Ly-6C^hi^-cells relative to the myeloid component at the onset of the clinical symptoms was inversely correlated with the SI. **e** Representative flow cytometry plots showing the percentage of Ly-6C^hi^-myeloid cells at the peak of the symptoms in EAE mice with a severe (left panel) or mild (right panel) clinical course. **f** The abundance of circulating Ly-6C^hi^-cells at the peak of the disease was directly correlated with the percentage of symptom recovery. Data from **a**, **b** are representative of seven independent experiments, *N* = 21 mice. For the correlation analysis, *N* = 16 mice

To evaluate the relationship between M-MDSCs and the future disease progression, we explore the relationship of their abundance in the blood and the clinical course in EAE mice. Given the lack of specific markers to distinguish Ly-6C^hi^-inflammatory monocytes from M-MDSCs according to the phenotype, the main characteristic that defines M-MDSCs is their ability to inhibit immune responses [44]. Circulating Ly-6C^hi^-cells isolated from the peripheral blood at disease onset were able to clearly reduce the proliferation of CD4^+^, CD8^+^ T cells, and B cells (identified as CD11b^−^/CD3^−^ cells) after 48 h in co-culture with MOG-stimulated splenocytes (Fig. 5a, b; Suppl. Fig. 2a). To avoid the putative immunosuppressive effect exerted by endogenous Ly-6C^hi^ cells over CD4^+^, CD8^+^ T cells and B cells, the same proliferation assays were carried out in the absence of Ly-6C^hi^ cells in the MOG-stimulated splenocytes. Again, circulating Ly-6C^hi^ cells exerted a strong immunosuppressive activity over both CD4^+^ and CD8^+^ T cells together with a trend toward a reduction in B-cell proliferation (Suppl. Fig. 2b–d). These data corroborated that circulating Ly-6C^hi^-cells at disease onset not only presented the markers but also the suppressive activity of M-MDSCs.

After that, we evaluated the correlation between the abundance of Ly-6C^hi^-cells in the peripheral blood and the severity of EAE clinical course. We showed that the higher abundance of Ly-6C^hi^-cells at disease onset the milder clinical course according to the severity index (SI) [27] (Fig. 5c, d), the maximum (*r* = − 0.521; *p* < 0.05), and the accumulated clinical score (*r* = − 0.615; *p* < 0.05). To validate the effect of sex on the relationship between Ly-6C^hi^-cells and the future disease severity, the same analysis was carried out in males with the EAE showing a similar correlation with the SI (Suppl. Fig. 3a, b). We also addressed the discriminating power of Ly-6C^hi^-cells at onset to assess the risk of developing mild or severe EAE clinical course according to the different parameters analyzed. Ly-6C^hi^-cells/myeloid cells presented a modest discriminating power to assess the risk of being mild/severe EAE mice based on the median value of the maximum clinical score at peak (AUC—area under the curve—0.800; 95% CI—confidence interval—0.514–1.026; *p* = 0.061) and the median value of the accumulated clinical score (AUC 0.813, 95% CI 0.571–1.054; *p* < 0.05). By contrast, Ly-6C^hi^-cells/myeloid cells presented the highest discriminatory power when classifying mild or severe EAE mice using the median of the SI (AUC 0.964, 95% CI 0.875–1.052; *p* < 0.01). In fact, we established cut-off values for the percentages of Ly6C^hi^-cells/myeloid cells, showing that values higher than 6.83% (100% specificity, 95% CI 59.04–100.0%; 77.78% sensitivity, 95% CI 39.99–97.19%) and lower than 5.47% (100% specificity, 95% CI 66.37–100.0%; 85.71% sensitivity, 95% CI 42.13–99.64%) at onset will be indicative of a future mild or severe EAE courses, respectively. In summary, these results suggested that circulating Ly-6C^hi^-cells at the EAE onset are strongly related to the future clinical course severity.

To check whether circulating Ly-6C^hi^-cells would also be useful to reveal the recovery of EAE mice, we evaluated the relationship between the abundance of these cells at the peak and clinical remission. We observed that the higher level of Ly-6C^hi^-cells at the peak of clinical course, the faster (recovered clinical score/days elapsed from peak to chronification; *r* = 0.652, *p* < 0.01) and greater % of recovery of EAE mice (Fig. 5f, g). Furthermore, the higher level of circulating Ly-6C^hi^-cells at the peak of the clinical course also showed a direct correlation with a higher % of recovery of male EAE mice (Suppl. Fig. 3c, d).

On the other hand, the level of Ly-6C^hi^-cells/myeloid cells did not discriminate the risk of developing mild/severe course in EAE mice at the end of the recovery phase based on the median value of the recovery index (AUC 0.730, 95% CI 0.435–0.970; *p* = 0.172) and the median value (67%) of the clinical score recovery (AUC 0.781, 95% CI 0.542–1.020; *p* = 0.058). However, the abundance of Ly-6C^hi^-cells at the peak showed the highest discriminatory power using 50% as recovery threshold (AUC 0.948, 95% CI 0.836–1.061; *p* < 0.05), establishing a cut-off value of 5.24% to be indicative of a mild/severe EAE recovery course. As such, values higher than the cut-off showed 100% specificity (95% CI 29.24–100.0%) and 92.31% sensitivity (95% CI 63.97–99.81%), whereas values below the same cut-off showed a 92.31% specificity (95% CI 63.97–99.81%) and 100% sensitivity (95% CI 29.24–100.0%).

These data reinforced the idea of the strong relationship between the level of circulating immunosuppressive Ly-6C^hi^-cells and the future clinical course severity and recovery extent after the peak of the symptoms in a sex-independent manner.

### Circulating Ly-6C^hi^-cells at the onset of the EAE are indicative of less CNS damage

**Fig. 6:**
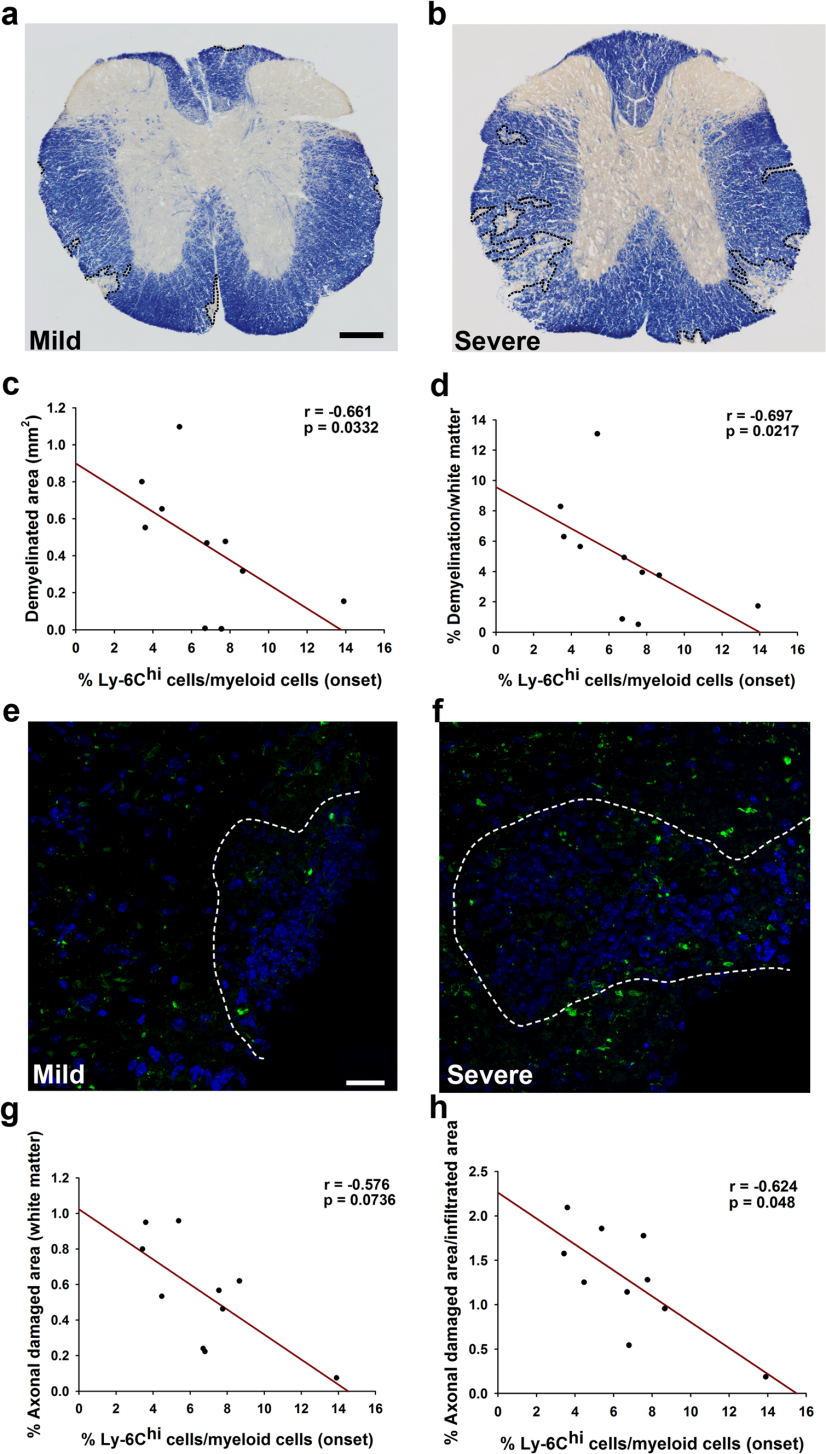
The Ly-6C^hi^-cell content in the peripheral blood at the onset of symptoms is indicative of lower CNS damage. Representative panoramic views of myelin staining with eriochrome cyanine of the spinal cord from a mild (**a**) or a severe (**b**) EAE mouse. The abundance of Ly-6C^hi^-cells at the onset of the clinical course was inversely correlated with the demyelinated area (**c**, **d**). SMI-32 staining showed more axonal damage in the spinal cord from severe (**f**) than mild (**e**) EAE mice. Ly-6C^hi^-cell abundance at the onset of the disease was inversely correlated with the extent of axonal damage (**g**, **h**). *N* = 10 mice. Scale bar: **a**, **b** = 100 µm; **e**, **f** = 25 µm

A second cohort of 10 EAE animals was used to analyze the correlation between Ly-6C^hi^-cells in the peripheral blood at EAE onset and the spinal cord pathology at the peak of disease. We first confirmed the correlation between Ly-6C^hi^-cells in the peripheral blood at the onset of the disease and the SI (*r* = − 0.726; *p* < 0.05). A reduced demyelination was observed in EAE mice with mild clinical course (Fig. 6a, b). A higher abundance of Ly-6C^hi^-cells was related to smaller areas of demyelination and the percentage it represented within the white matter (Fig. 6c, d). In parallel, axonal damage was less prominent in mild EAE mice (Fig. 6e, f) and the higher abundance of Ly-6C^hi^-cells at disease onset was related to the lower degree of axonal damage within the white matter (Fig. 6g) or within the infiltrated area (Fig. 6h).

In conclusion, the higher Ly-6C^hi^-cell content in blood at disease onset pointed to a lower degree of CNS affectation at the peak of the disease, indicating that the levels of circulating Ly-6C^hi^-cells are closely related to the future disease evolution and CNS damage extent in EAE mice.

### M-MDSCs in MS patients are indicative of a better relapse recovery

**Fig. 7:**
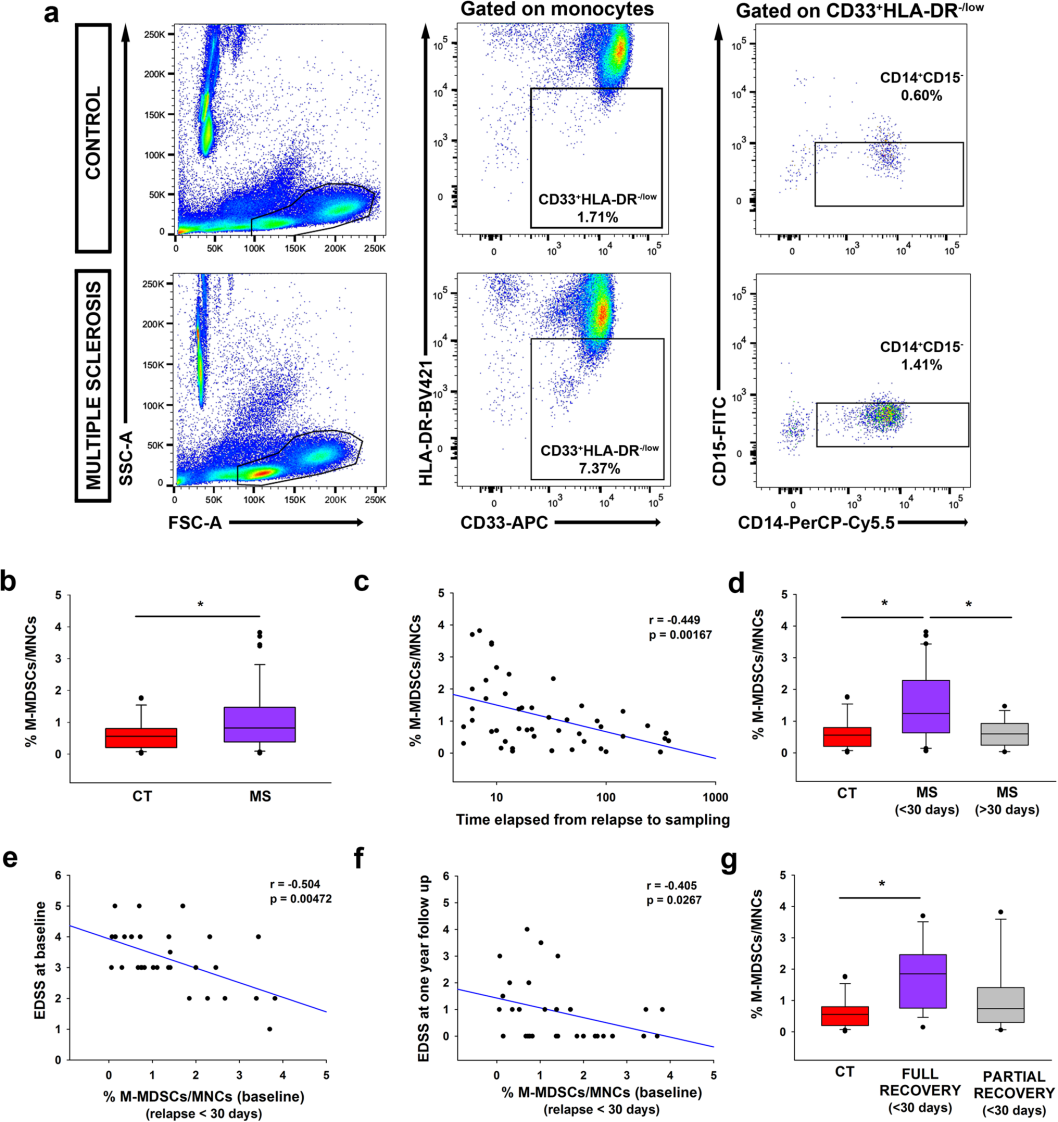
The abundance of M-MDSCs in the peripheral blood of MS patients is indicative of a better relapse recovery in MS. **a** Representative flow cytometry plots for M-MDSCs in human PBMC samples. In both control and MS patients, live MNCs were gated after removing cellular aggregates and death cells based in ZombiNIR expression. Next, immature myeloid cells were gated from monocyte subpopulation as CD33^+^HLA-DR^−/low^ populations. CD33^+^HLA-DR^−/low^-cells were assessed for CD14 and CD15 expression to identify the M-MDSC subset defined as CD14^+^CD15^−^ (in this panel, the percentage of M-MDSCs refers to MNCs). **b** The abundance of M-MDSCs measured in MS patients at an early stage of their disease course was significantly higher than controls. **c** The M-MDSC load in human peripheral blood was inversely correlated with the time elapsed from the first relapse to sampling. **d** M-MDSCs were more abundant in MS patients at the time of their first referred relapse (≤ 30 days after the relapse) than in controls and MS patients in remission (> 30 days after the relapse). In the sub-cohort of MS patients in relapse, the abundance of M-MDSCs at baseline was inversely correlated with the EDSS at baseline (**e**) and with the EDSS 1 year later (**f**). **g** Only those MS patients at relapse with full recovery (with an EDSS of 0 at 12 months) had a higher proportion of M-MDSCs than healthy controls. Control in **b** and **g** *N* = 26; MS in **b** and **c** *N* = 47; MS ≤ 30 days in **d**–**g** *N* = 30; MS > 30 days in **d** *N* = 17. Full recovery *N* = 15 and partial recovery *N* = 15 MS patients in **g**

Finally, we examined the predictive capacity of M-MDSCs in the blood of MS patients at an early time point in their clinical course. M-MDSCs were classified in the PBMCs of healthy controls (HCs) and MS patients as CD33^+^HLA-DR^−/low^CD14^+^CD15^−^-cells (Fig. 7a; gating strategy shown in Suppl Fig. 4) [2]. MS patients with a first clinical episode suggestive of MS in the last year were enrolled (Suppl.Table 1). M-MDSCs were significantly higher in MS patients than HCs (Fig. 7b). M-MDSC numbers were independent of the age or EDSS at baseline in MS patients (not shown). Interestingly, the higher M-MDSC abundance was related to shorter periods of time from relapse to sampling (Fig. 7c). Since symptoms occurring within a month after clinical manifestations were considered to be part of the same relapse [34], we split the patients into two groups: those whose blood was collected ≤ 30 days (relapse) or more than 30 days after relapse (remission; Suppl. Table 1). MS patients in relapse had a higher percentage of M-MDSCs than HCs and patients in remission (Fig. 7d), indicating that M-MDSCs were higher close to the inflammatory episode.

To establish the relationship between M-MDSCs and disease evolution, MS patients in relapse were 1-year follow-up (Suppl.Table 2). M-MDSC at baseline was independent of the age (*r* = − 0.0742, *p* = 0.619) or the number of Gd^+^ enhancing lesions (*r* = 0342, *p* = 0.826). Importantly, the higher M-MDSC abundance at baseline was associated with a lower EDSS at sampling and at 1-year follow-up (Fig. 7e, f). Interestingly, M-MDSCs were exclusively enriched at baseline in those MS patients with a full relapse recovery 1 year later (EDSS of 0 at 12 months) (*p* < 0.05: Fig. 7g). Conversely, the proportion of M-MDSCs at 1-year follow-up was independent of the EDSS at that time point, nor was there any difference in the proportion of M-MDSCs between fully or partially recovered patients (not shown).

In summary, these data indicate that the high M-MDSC content in MS patients close to relapse was associated with a less disability at the time of sampling and to a better recovery after 12 months of follow-up.

## Discussion

The data presented here show for the first time the presence of infiltrated myeloid cells exhibiting the complete M-MDSC phenotype in human tissue from MS patients, mainly in areas with a high inflammatory activity. Indeed, we detected a direct correlation between the lower abundance of M-MDSC putative cells in these areas and both the shorter age at death and disease duration in PPMS patients. Furthermore, we provided novel insights into the close relationship between immunosuppressive Ly-6C^hi^-cells/M-MDSCs and the future clinical course of the EAE model in mice and relapse recovery in MS subjects. Our results show that when there is a higher abundance of immunosuppressive Ly-6C^hi^-cells at the onset of EAE, the disease course will be milder. Interestingly, we translated these data into MS, showing that the abundance of M-MDSCs in blood samples from untreated patients at their first relapse is inversely correlated to the EDSS at baseline and after a 1-year follow-up with those who will exhibit a full recovery having a higher M-MDSC abundance than controls.

In our study, the characterization of M-MDSCs was based on the commonly accepted phenotypic markers used for their classification in blood and associated immune organs, which remains a challenge [44]. In all cases, M-MDSCs have been described in blood as CD14^+^HLA-DR^−/low^CD15^−^CD33^+^CD11b^+^, which completely fits with our observations in the human CNS. Furthermore, to shed further light on the origin of these cells, we took advantage of TMEM119 as the only microglia-specific marker in humans [39]. However, TMEM119 disappeared in activated microglia from the center of ALs and AILs, but remains at the edge of ALs [46]. Our data clearly demonstrated that cells with the M-MDSC phenotype found in the most peripheral zone of ALs did not express TMEM119, including those located within the CNS parenchyma and blood vessel-associated cells, which might have infiltrated into the CNS more recently. We cannot ensure the origin of cells with the phenotype of M-MDSCs in the center of ALs or within other white matter lesions. However, we did not observe any differences in morphology, distribution, or immunophenotype between patients, which leads us to consider that all cells with the same phenotype might be classified as M-MDSCs. Furthermore, there are no data on differences in the presence of the various M-MDSC markers in blood between MS patients [12]. Our data represent the closest approximation to date of the presence of cells with the phenotype of M-MDSCs in the brains of MS patients. However, to be considered an MDSC, a myeloid cell must not only exhibit the full phenotype described here, but also its own anti-proliferative activity. In one of the most extensive review on the classification of M-MDSCs [44], CXCR1 and CD84 appeared as the two new phenotypic markers associated with their immunosuppressive activity that have been added for the classification of M-MDSCs in cancer. CXCR1 is a chemokine receptor expressed on neutrophils and normally not associated with monocytes that has been described to be present in up to 20% of M-MDSCs, showing a reduced or absent immunosuppressive activity [25]. For these reasons, we consider CXCR1 to be a useless marker to validate the presence and activity of M-MDSCs in the context of MS tissue samples. In contrast, CD84 is present in a significant proportion of highly immunosuppressive M-MDSCs in the blood of cancer patients (between 60 and 95%) [19, 20], which is consistent with our observations in MS white matter lesions. Further studies are needed to fully understand the importance of this marker in the context of MS. To be absolutely certain that the cells described here were M-MDSCs, they would need to be isolated from MS patient biopsies with which to test their anti-proliferative activity on lymphoid cells, something which is beyond the technical scope of the present work.

Despite the enormous progress in developing DMTs for MS [24], a better understanding of the mechanisms driving the heterogeneity in the clinical course of the disease is crucial for the future prediction of disease progression, and the prompt and precise improvement of the disease by early and accurate treatments. The suppressive function of immunoregulatory cells such as Treg is closely related to pathological progression [10, 45]. Furthermore, previous studies into MS using human brain samples demonstrated that the neuronal pathological changes and inflammation are closely related in both SPMS and PPMS patients, as they both display similar inflammatory activity [3, 11, 23]. These observations highlight the role of the immune response in a subset of progressive MS patients, as confirmed by the approval of the immunomodulatory treatments, such as ocrelizumab or siponimod as EMA/FDA-approved drugs for PPMS and SPMS, respectively [14, 33]. For the first time, we show a correlation between the regulatory myeloid cells (i.e., M-MDSCs) and the disease duration of PPMS. In our studies, the lower the abundance of the cells with the phenotype of M-MDSCs in areas with a high inflammatory activity, the shorter the disease course of PPMS patients. Furthermore, the ratio between CD14^+^HLA-DR^hi^ pro-inflammatory cells and M-MDSCs was inversely correlated with the disease duration in PPMS, suggesting that the dampened immunoregulatory context might worsen the disease progression in these patients. Since all PPMS samples in the UK MS and Dame Ingrid V Allen tissue banks were collected before therapies for PPMS were available, the M-MDSC distribution in these patients should reflect the distribution of immune cells at the end of the unmodified natural history of the disease. This point is supported by the identification of a similar direct correlation between the density of the cells resembling M-MDSCs and the age of PPMS patients. In this sense, the low density of M-MDSCs in high inflammatory regions of PPMS patients with shorter disease durations reinforces the notion that abnormalities in regulatory mechanisms may affect the clinical course. Our results may open the door for the future use of M-MDSCs as biomarkers of more benign/severe PPMS courses, which would have important consequences for the design of new clinical trials in this particular patients’ group as well as treatment decision-making by patients. Interestingly, the density of M-MDSC-like cells in demyelinated lesions is not correlated with disease severity in SPMS. The unexpected difference in the correlation in PPMS and SPMS patients of cells with the MDSC phenotype with disease severity may be explained in different ways. In blood, M-MDSCs are less abundant and immunosuppressive in SPMS patients than in RRMS patients and fail to suppress T-cell proliferation [12]. Thus, the lack of correlation between the M-MDSCs and the duration of the SPMS clinical courses might be due to weaker immunosuppression or to exhaustion from repeated inflammatory attacks. Alternatively, the final distribution and probably the activity state of M-MDSCs in the CNS of SPMS may be the result of their modification after different therapeutic treatments during the relapsing–remitting phase. Indeed, this has been described for cells of the innate immune response in the blood of MS patients [1, 27], including MDSCs [2, 48]. Due to the impossibility of validating the immunosuppressive activity of our M-MDSC-like cells, we cannot rule out the possibility that they are infiltrating myeloid cells of another type whose phenotype is modulated according to the inflammatory state of each patient. In SPMS, M-MDSC-like cells are more likely to show a stage-dependent phenotypic shift of myeloid cells as a consequence of newly induced classical active lesions, whereas in PPMS, it could be a more homogeneous stage of activity in chronic active lesions. Therefore, the findings might rather show a stage-dependent phenotypic change of myeloid cells rather than a population of suppressor cells. Finally, to have a cohort of PPMS patients with long clinical courses, our study cohort has an overrepresentation of PPMS patients with early onsets and even longer clinical courses than those of SPMS patients. This could imply an inflammatory bias in patients with PPMS compared to SPMS, which could be an alternative explanation for the correlation between MDSC-like cells only in this progressive form of the disease.

The data presented here show an inverse correlation between the levels of immunosuppressive Ly-6C^hi^-cells in the peripheral blood of mice at the onset of the clinical signs and the severity of the disease course in a sex-independent manner. After analyzing the spinal cord of EAE mice, we observe that the abundance of Ly-6C^hi^-cells at disease onset inversely correlated with CNS damage, which included less destruction of myelin and axonal damage. Murine M-MDSCs from immune organs and infiltrated spinal cord of EAE mice share the same immunophenotype than the so-called Ly-6C^hi^-cells [48, 49]. Apart from the phenotype, the main characteristic that defines M-MDSCs is their ability to inhibit immune responses [44]. For this reason, previous to their putative use as biomarkers of disease severity, we probed that Ly-6C^hi^-cells isolated from the whole blood at the onset of the symptoms exerted and important immunosuppressive function activity over T and B cells. Our results indicate that immunosuppressive Ly-6C^hi^-cells present both the phenotype and activity to be fully considered M-MDSCs.

There are controversial data, indicating that Ly-6C^hi^-cells may play pro-inflammatory or immunosuppressive roles in the different phases of the EAE clinical course [15, 49]. It has been described that almost 98% of the Arg-I^+^-anti-inflammatory cells that infiltrate the CNS at the peak of the clinical course were CCR2^+^-invading cells [21]. Furthermore, 50% of infiltrating Arg-I^+^ macrophages in the CNS at the peak derived from pro-inflammatory monocyte-derived iNOS-expressing cells invading this area at the onset of the clinical course [8]. Taking together, it could be thought that the higher Ly-6C^hi^ peripheral blood cell content at the onset of the disease, the higher density of anti-inflammatory cells found at the peak of the symptoms. Previous studies illustrated how the anti-inflammatory environment promoted by the anti-inflammatory polarization of microglia/macrophages may help ameliorate EAE progression and promote remyelination [30]. Furthermore, Ly-6C^hi^-cells are able to change their activity by the shift from pro-inflammatory to a clear anti-inflammatory activity after IFN-β treatment [29], suggesting that the presence of this malleable cell type at the onset of EAE should result in a higher abundance of immunosuppressive M-MDSCs at the peak of the disease. It is beyond the scope of this work to determine whether these Ly-6C^hi^-cells found at disease onset are the same cells as those detected at the disease peak. However, the crucial role of pro-inflammatory Ly-6C^hi^-cells in the effective repair at later stages of chronic inflammatory pathologies was reported recently [37]. In this sense, the stronger presence of immunosuppressive Ly-6C^hi^-cells at the onset of the clinical signs might help enhance the repair mechanisms involved in the later phases of the clinical course of EAE. In support of this, it was recently described that M-MDSCs promote remyelination in EAE by enhancing oligodendrocyte precursor cell (OPC) survival, proliferation, and differentiation [28], suggesting the crucial role of M-MDSCs not only in resolving inflammation but also in promoting tissue regeneration. However, the protective role of M-MDSCs on remyelination may not be ruled out by indirect function by promoting the increase of Treg population [35].

Finally, we found that the abundance of M-MDSCs in blood samples from untreated MS patients at relapse correlates with a lower EDSS at baseline and after a 1-year follow-up. The analysis of this regulatory cell population suggests a role of M-MDSCs as a future biomarker of MS clinical progression, though the stratification based on DMTs needs for further validation in independent and larger cohorts. It is important to note that MDSCs were recently shown to have certain relevance in terms of the recovery of the MS clinical course, whereby the expansion of MDSCs in glucocorticoid-treated MS patients may help alleviate the acute phase of the disease [47]. Furthermore, in a recent work of our group, it has been shown that circulating Ly-6C^hi^ cells have the ability to help classify EAE mice into responders and non-responders to fingolimod, which, as in the current work, had a translational impact in MS patients [1]. However, these promising results about the close relationship between MDSCs and disease severity in MS should be validated in a long-standing and larger cohort of MS patients.

In summary, the current results from human samples together with the observations in the EAE model suggest that cells with the full phenotype of M-MDSCs, and suppressor activity in the case of EAE, are strongly related to past or future clinical course of the disease. This opens the door to their study as a future biomarker for disease severity which needs for further validation. As such, the therapeutic use of M-MDSCs could be considered as a strategy for improving immunoregulatory mechanisms as well as myelin repair to alleviate the disease course.

## Supplementary Information

Below is the link to the electronic supplementary material.

Supplementary file1 (PDF 836 KB)

Supplementary file2 (DOCX 16 KB)

Supplementary file3 (XLSX 37 KB)

## Data Availability

All data supporting the fndings of this study are available within the paper and as raw data in Supplementary Information. Extra information about the data is available from the corresponding author upon reasonable request.

## References

[1] Camacho-Toledano C, Machín-Díaz I, Calahorra L, Cabañas-Cotillas M, Otaegui D, Castillo-Triviño T, Villar LM, Costa-Frossard L, Comabella M, Midaglia L, García-Domínguez JM, García-Arocha J, Ortega MC, Clemente D (2022). Peripheral myeloid-derived suppressor cells are good biomarkers of the efficacy of fingolimod in multiple sclerosis. J Neuroinflamm.

[2] Cantoni C, Cignarella F, Ghezzi L, Mikesell B, Bollman B, Berrien-Elliott MM, Ireland AR, Fehniger TA, Wu GF, Piccio L (2017). Mir-223 regulates the number and function of myeloid-derived suppressor cells in multiple sclerosis and experimental autoimmune encephalomyelitis. Acta Neuropathol.

[3] Choi SR, Howell OW, Carassiti D, Magliozzi R, Gveric D, Muraro PA, Nicholas R, Roncaroli F, Reynolds R (2012). Meningeal inflammation plays a role in the pathology of primary progressive multiple sclerosis. Brain.

[4] Clemente D, Ortega MC, Arenzana FJ, de Castro F (2011). FGF-2 and anosmin-1 are selectively expressed in different types of multiple sclerosis lesions. J Neurosci.

[5] Dendrou CA, Fugger L, Friese MA (2015). Immunopathology of multiple sclerosis. Nat Rev Immunol.

[6] Dolcetti L, Peranzoni E, Ugel S, Marigo I, Gomez AF, Mesa C, Geilich M, Winkels G, Traggiai E, Casati A, Grassi F, Bronte V (2010). Hierarchy of immunosuppressive strength among myeloid-derived suppressor cell subsets is determined by GM-CSF. Eur J Immunol.

[7] Filippi M, Rocca MA, Ciccarelli O, De Stefano N, Evangelou N, Kappos L, Rovira A, Sastre-Garriga J, Tintorè M, Frederiksen JL, Gasperini C, Palace J, Reich DS, Banwell B, Montalban X, Barkhof F (2016). MRI criteria for the diagnosis of multiple sclerosis: MAGNIMS consensus guidelines. Lancet Neurol.

[8] Giles DA, Washnock-Schmid JM, Duncker PC, Dahlawi S, Ponath G, Pitt D, Segal BM (2018). Myeloid cell plasticity in the evolution of central nervous system autoimmunity. Ann Neurol.

[9] Gjelstrup MC, Stilund M, Petersen T, Møller HJ, Petersen EL, Christensen T (2018). Subsets of activated monocytes and markers of inflammation in incipient and progressed multiple sclerosis. Immunol Cell Biol.

[10] Haas J, Hug A, Viehöver A, Fritzsching B, Falk CS, Filser A, Vetter T, Milkova L, Korporal M, Fritz B, Storch-Hagenlocher B, Krammer PH, Suri-Payer E, Wildemann B (2005). Reduced suppressive effect of CD4+CD25high regulatory T cells on the T cell immune response against myelin oligodendrocyte glycoprotein in patients with multiple sclerosis. Eur J Immunol.

[11] Howell OW, Reeves CA, Nicholas R, Carassiti D, Radotra B, Gentleman SM, Serafini B, Aloisi F, Roncaroli F, Magliozzi R, Reynolds R (2011). Meningeal inflammation is widespread and linked to cortical pathology in multiple sclerosis. Brain.

[12] Iacobaeus E, Sugars RV, Törnqvist Andrén A, Alm JJ, Qian H, Frantzen J, Newcombe J, Alkass K, Druid H, Bottai M, Röyttä M, Le Blanc K (2017). Dynamic changes in brain mesenchymal perivascular cells associate with multiple sclerosis disease duration, active inflammation, and demyelination. Stem Cells Transl Med.

[13] Ioannou M, Alissafi T, Lazaridis I, Deraos G, Matsoukas J, Gravanis A, Mastorodemos V, Plaitakis A, Sharpe A, Boumpas D, Verginis P (2012). Crucial role of granulocytic myeloid-derived suppressor cells in the regulation of central nervous system autoimmune disease. J Immunol.

[14] Kappos L, Bar-Or A, Cree BAC, Fox RJ, Giovannoni G, Gold R, Vermersch P, Arnold DL, Arnould S, Scherz T, Wolf C, Wallström E, Dahlke F, Achiron A (2018). Siponimod versus placebo in secondary progressive multiple sclerosis (EXPAND): a double-blind, randomised, phase 3 study. Lancet.

[15] King IL, Dickendesher TL, Segal BM (2009). Circulating Ly-6C + myeloid precursors migrate to the CNS and play a pathogenic role during autoimmune demyelinating disease. Blood.

[16] Kirschbaum K, Sonner JK, Zeller MW, Deumelandt K, Bode J, Sharma R, Krüwel T, Fischer M, Hoffmann A, Da Silva MC, Muckenthaler MU, Wick W, Tews B, Chen JW, Heiland S, Bendszus M, Platten M, Breckwoldt MO (2016). In vivo nanoparticle imaging of innate immune cells can serve as a marker of disease severity in a model of multiple sclerosis. Proc Natl Acad Sci U S A.

[17] Knier B, Hiltensperger M, Sie C, Aly L, Lepennetier G, Engleitner T, Garg G, Muschaweckh A, Mitsdörffer M, Koedel U, Höchst B, Knolle P, Gunzer M, Hemmer B, Rad R, Merkler D, Korn T (2018). Myeloid-derived suppressor cells control B cell accumulation in the central nervous system during autoimmunity. Nat Immunol.

[18] Kuhlmann T, Ludwin S, Prat A, Antel J, Brück W, Lassmann H (2017). An updated histological classification system for multiple sclerosis lesions. Acta Neuropathol.

[19] Lewinsky H, Barak AF, Huber V, Kramer MP, Radomir L, Sever L, Orr I, Mirkin V, Dezorella N, Shapiro M, Cohen Y, Shvidel L, Seiffert M, Herishanu Y, Becker-Herman S, Shachar I (2018). CD84 regulates PD-1/PD-L1 expression and function in chronic lymphocytic leukemia. J Clin Invest.

[20] Lewinsky H, Gunes EG, David K, Radomir L, Kramer MP, Pellegrino B, Perpinial M, Chen J, He TF, Mansour AG, Teng KY, Bhattacharya S, Caserta E, Troadec E, Lee P, Feng M, Keats J, Krishnan A, Rosenzweig M, Yu J, Caligiuri MA, Cohen Y, Shevetz O, Becker-Herman S, Pichiorri F, Rosen S, Shachar I (2021). CD84 is a regulator of the immunosuppressive microenvironment in multiple myeloma. JCI Insight.

[21] Locatelli G, Theodorou D, Kendirli A, Jordão MJC, Staszewski O, Phulphagar K, Cantuti-Castelvetri L, Dagkalis A, Bessis A, Simons M, Meissner F, Prinz M, Kerschensteiner M (2018). Mononuclear phagocytes locally specify and adapt their phenotype in a multiple sclerosis model. Nat Neurosci.

[22] Luchetti S, Fransen NL, van Eden CG, Ramaglia V, Mason M, Huitinga I (2018). Progressive multiple sclerosis patients show substantial lesion activity that correlates with clinical disease severity and sex: a retrospective autopsy cohort analysis. Acta Neuropathol.

[23] Magliozzi R, Howell OW, Nicholas R, Cruciani C, Castellaro M, Romualdi C, Rossi S, Pitteri M, Benedetti MD, Gajofatto A, Pizzini FB, Montemezzi S, Rasia S, Capra R, Bertoldo A, Facchiano F, Monaco S, Reynolds R, Calabrese M (2018). Inflammatory intrathecal profiles and cortical damage in multiple sclerosis. Ann Neurol.

[24] Martin R, Sospedra M, Rosito M, Engelhardt B (2016). Current multiple sclerosis treatments have improved our understanding of MS autoimmune pathogenesis. Eur J Immunol.

[25] Mastio J, Condamine T, Dominguez G, Kossenkov AV, Donthireddy L, Veglia F, Lin C, Wang F, Fu S, Zhou J, Viatour P, Lavilla-Alonso S, Polo AT, Tcyganov EN, Mulligan C, Nam B, Bennett J, Masters G, Guarino M, Kumar A, Nefedova Y, Vonderheide RH, Languino LR, Abrams SI, Gabrilovich DI (2019). Identification of monocyte-like precursors of granulocytes in cancer as a mechanism for accumulation of PMN-MDSCs. J Exp Med.

[26] Mecha M, Feliú A, Machín I, Cordero C, Carrillo-Salinas F, Mestre L, Hernández-Torres G, Ortega-Gutiérrez S, López-Rodríguez ML, de Castro F, Clemente D, Guaza C (2018). 2-AG limits Theiler’s virus induced acute neuroinflammation by modulating microglia and promoting MDSCs. Glia.

[27] Melero-Jerez C, Alonso-Gómez A, Moñivas E, Lebrón-Galán R, Machín-Díaz I, de Castro F, Clemente D (2020). The proportion of myeloid-derived suppressor cells in the spleen is related to the severity of the clinical course and tissue damage extent in a murine model of multiple Sclerosis. Neurobiol Dis.

[28] Melero-Jerez C, Fernández-Gómez B, Lebrón-Galán R, Ortega MC, Sánchez-de Lara I, Ojalvo AC, Clemente D, de Castro F (2021). Myeloid-derived suppressor cells support remyelination in a murine model of multiple sclerosis by promoting oligodendrocyte precursor cell survival, proliferation, and differentiation. Glia.

[29] Melero-Jerez C, Suardíaz M, Lebrón-Galán R, Marín-Bañasco C, Oliver-Martos B, Machín-Díaz I, Fernández Ó, de Castro F, Clemente D (2019). The presence and suppressive activity of myeloid-derived suppressor cells are potentiated after interferon-β treatment in a murine model of multiple sclerosis. Neurobiol Dis.

[30] Miron VE, Boyd A, Zhao JW, Yuen TJ, Ruckh JM, Shadrach JL, Van Wijngaarden P, Wagers AJ, Williams A, Franklin RJM, Ffrench-Constant C (2013). M2 microglia and macrophages drive oligodendrocyte differentiation during CNS remyelination. Nat Neurosci.

[31] Moliné-Velázquez V, Cuervo H, Vila-Del Sol V, Ortega MC, Clemente D, De Castro F (2011). Myeloid-derived suppressor cells limit the inflammation by promoting T lymphocyte apoptosis in the spinal cord of a murine model of multiple sclerosis. Brain Pathol.

[32] Moliné-Velázquez V, Ortega MC, Vila-del Sol V, Melero-Jerez C, de Castro F, Clemente D (2014). Myeloid-derived suppressor cells are key elements for symptom recovery in a murine model of multiple sclerosis. J Neuroimmunol.

[33] Montalban X, Hauser SL, Kappos L, Arnold DL, Bar-Or A, Comi G, de Seze J, Giovannoni G, Hartung H-P, Hemmer B, Lublin F, Rammonah KW, Selmaj K, Traboulsee A, Sauter A, Masterman D, Fontoura P, Belachew S, Garren H, Mairon N, Chin P, Wolinsky JS (2017). Ocrelizumab versus placebo in primary progressive multiple sclerosis. N Engl J Med.

[34] Novotna M, Paz Soldán MM, Zeid NA, Kale N, Tutuncu M, Crusan DJ, Atkinson EJ, Siva A, Keegan BM, Pirko I, Pittock SJ, Lucchinetti CF, Noseworthy JH, Weinshenker BG, Rodriguez M, Kantarci OH (2015). Poor early relapse recovery affects onset of progressive disease course in multiple sclerosis. Neurology.

[35] Pan PY, Ma G, Weber KJ, Ozao-Choy J, Wang G, Yin B, Divino CM, Chen SH (2010). Immune stimulatory receptor CD40 is required for T cell suppression and T regulatory cell activation mediated by myeloid-derived suppressor cells in cancer. Cancer Res.

[36] Reich DS, Lucchinetti CF, Calabresi PA (2018). Multiple sclerosis. N Engl J Med.

[37] Rizzo G, Di Maggio R, Benedetti A, Morroni J, Bouche M, Lozanoska-Ochser B (2020). Splenic Ly6Chi monocytes are critical players in dystrophic muscle injury and repair. JCI Insight.

[38] Ruifrok AC, Johnston DA (2001). Quantification of histochemical staining by color deconvolution. Anal Quant Cytol Histol.

[39] Satoh J, Kino Y, Asahina N, Takitani M, Miyoshi J, Ishida T, Saito Y (2016). TMEM119 marks a subset of microglia in the human brain. Neuropathology.

[40] Sintes J, Romero X, de Salort J, Terhorst C, Engel P (2010). Mouse CD84 is a pan-leukocyte cell-surface molecule that modulates LPS-induced cytokine secretion by macrophages. J Leukoc Biol.

[41] Thompson AJ, Baranzini SE, Geurts J, Hemmer B, Ciccarelli O (2018). Multiple sclerosis. Lancet.

[42] Tintore M, Vidal-Jordana A, Sastre-Garriga J (2019). Treatment of multiple sclerosis—success from bench to bedside. Nat Rev Neurol.

[43] Veglia F, Perego M, Gabrilovich D (2018). Myeloid-derived suppressor cells coming of age review-article. Nat Immunol.

[44] Veglia F, Sanseviero E, Gabrilovich DI (2021). Myeloid-derived suppressor cells in the era of increasing myeloid cell diversity. Nat Rev Immunol.

[45] Viglietta V, Baecher-Allan C, Weiner HL, Hafler DA (2004). Loss of functional suppression by CD4+CD25+ regulatory T cells in patients with multiple sclerosis. J Exp Med.

[46] Van Wageningen TA, Vlaar E, Kooij G, Jongenelen CAM, Geurts JJG, Van Dam AM (2019). Regulation of microglial TMEM119 and P2RY12 immunoreactivity in multiple sclerosis white and grey matter lesions is dependent on their inflammatory environment. Acta Neuropathol Commun.

[47] Wang Z, Zheng G, Li G, Wang M, Ma Z, Li H, Wang XY, Yi H (2020). Methylprednisolone alleviates multiple sclerosis by expanding myeloid-derived suppressor cells via glucocorticoid receptor β and S100A8/9 up-regulation. J Cell Mol Med.

[48] Zhu B, Bando Y, Xiao S, Yang K, Anderson AC, Kuchroo VK, Khoury SJ (2007). CD11b+Ly-6C hi suppressive monocytes in experimental autoimmune encephalomyelitis. J Immunol.

[49] Zhu B, Kennedy JK, Wang Y, Sandoval-Garcia C, Cao L, Xiao S, Wu C, Elyaman W, Khoury SJ (2011). Plasticity of Ly-6C hi myeloid cells in T cell regulation. J Immunol.

