# Supplementary material for: Central and peripheral myeloid-derived suppressor cell-like cells are closely related to the clinical severity of multiple sclerosis
Source: Acta Neuropathol. 2023 May 27;146(2):263–82. doi: 10.1007/s00401-023-02593-x (PMC10329064; doi:10.1007/s00401-023-02593-x)
Supplement: Supplementary file 1 — Supplementary file1 (PDF 836 KB) [file 401_2023_2593_MOESM1_ESM.pdf]

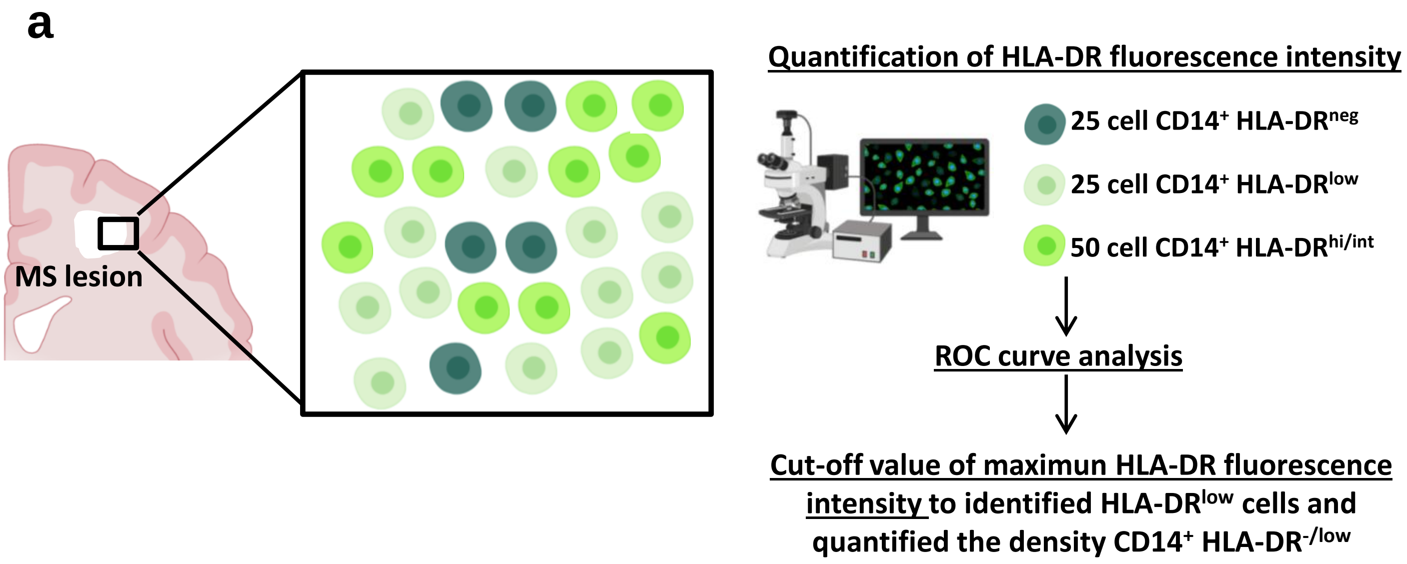

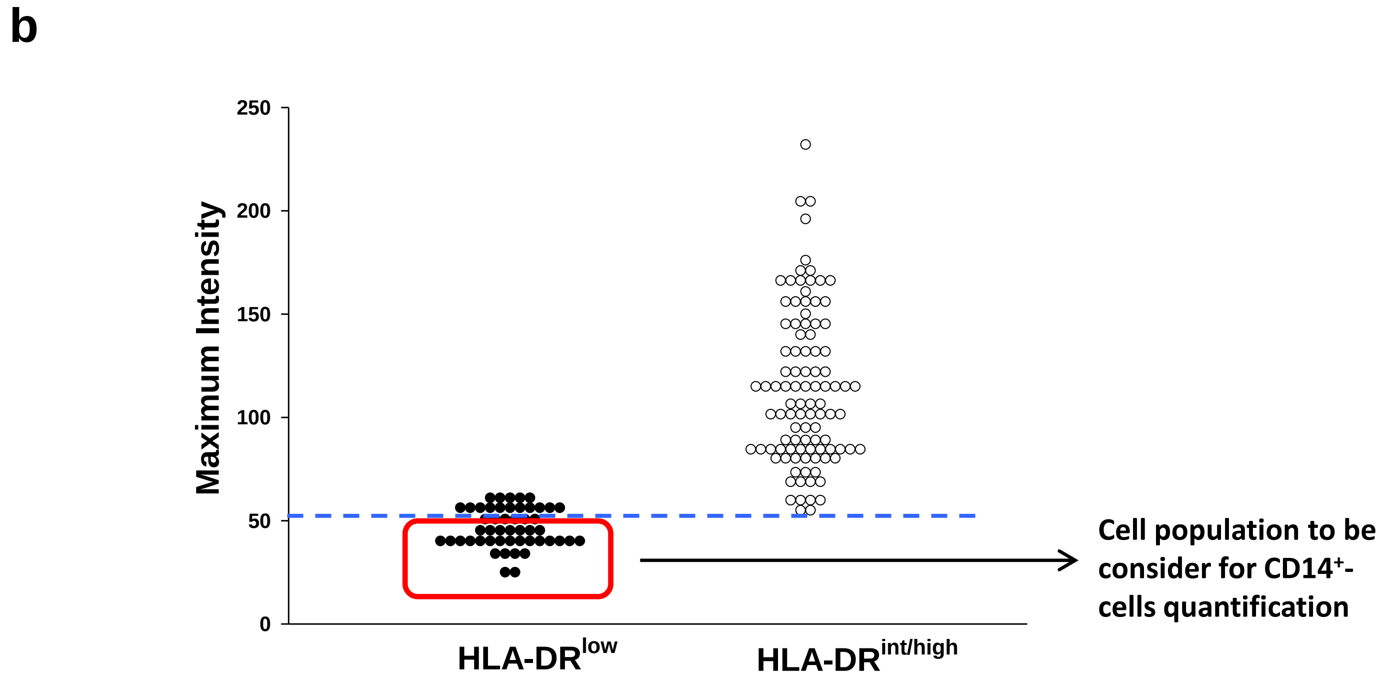

Suppl. Figure 1

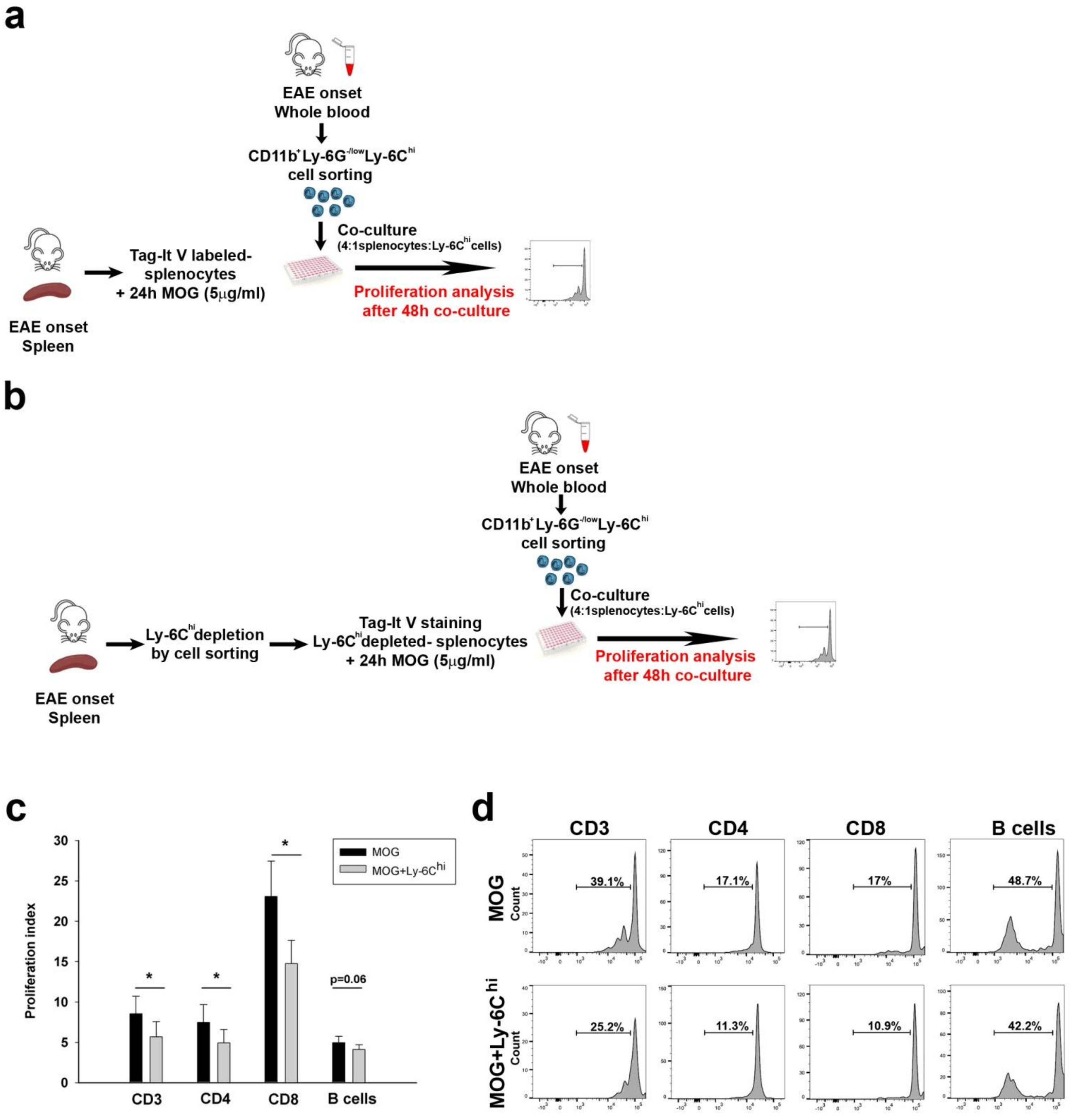
a
EAE onset
Whole blood
CD11b+Ly-6G-/lowLy-6Chi
cell sorting
Co-culture
(4:1splenocytes:Ly-6Chicells)
Tag-It V labeled-
splenocytes
+ 24h MOG (5μg/ml)
Proliferation analysis
after 48h co-culture
EAE onset
Spleen
b
EAE onset
Whole blood
CD11b+Ly-6G-/lowLy-6Chi
cell sorting
Co-culture
(4:1splenocytes:Ly-6Chicells)
Ly-6Chidepletion
by cell sorting
Tag-It V staining
Ly-6Chidepleted- splenocytes
+ 24h MOG (5μg/ml)
Proliferation analysis
after 48h co-culture
EAE onset
Spleen
c
MOG
MOG+Ly-6Chi
Proliferation index
30
25
20
15
10
5
0
*
*
*
p=0.06
CD3
CD4
CD8
B cells
d
CD3
CD4
CD8
B cells
MOG
Count
39.1%
17.1%
17%
48.7%
MOG+Ly-6Chi
Count
25.2%
11.3%
10.9%
42.2%

Suppl. Fig. 2

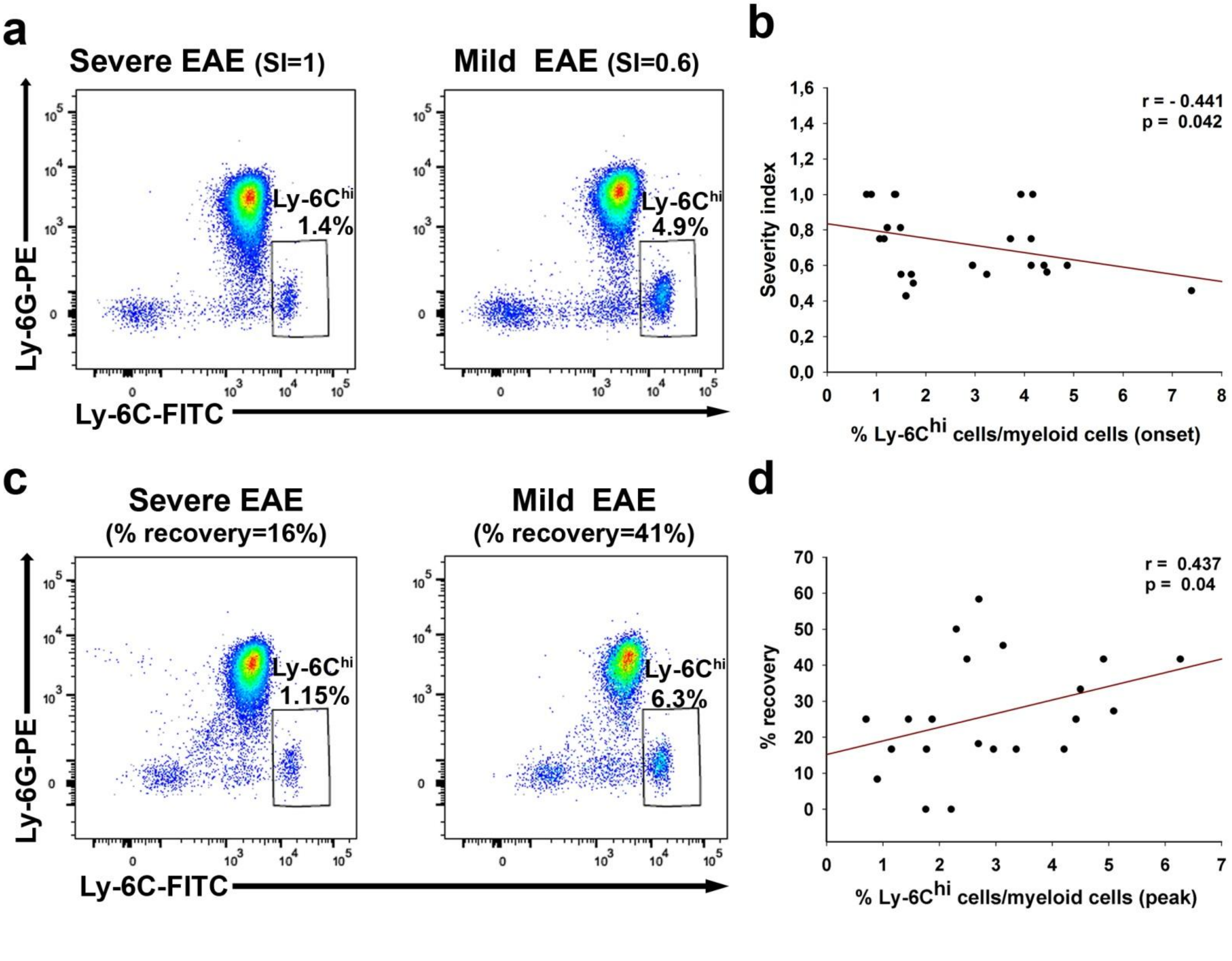
a
Severe EAE (SI=1)
Mild EAE (SI=0.6)
Ly-6G-PE
Ly-6C-FITC
Ly-6Chi 1.4%
Ly-6Chi 4.9%
b
Severity index
% Ly-6Chi cells/myeloid cells (onset)
r = - 0.441
p = 0.042
c
Severe EAE (% recovery=16%)
Mild EAE (% recovery=41%)
Ly-6G-PE
Ly-6C-FITC
Ly-6Chi 1.15%
Ly-6Chi 6.3%
d
% recovery
% Ly-6Chi cells/myeloid cells (peak)
r = 0.437
p = 0.04

Suppl. Fig. 3

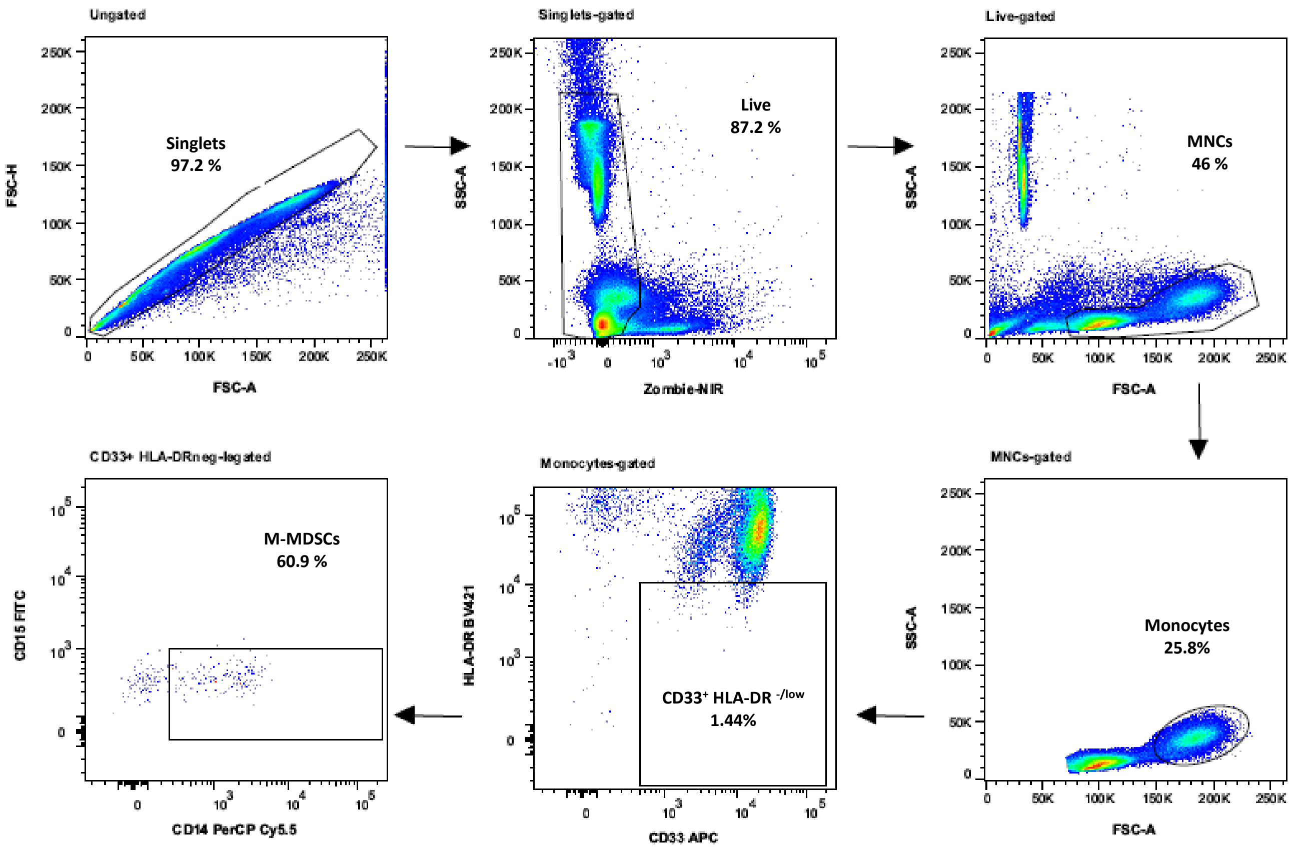
Ungated
Singlets
97.2 %
FSC-H
FSC-A
Singlets-gated
Live
87.2 %
SSC-A
Zombie-NIR
Live-gated
MNCs
46 %
SSC-A
FSC-A
MNCs-gated
Monocytes
25.8%
SSC-A
FSC-A
Monocytes-gated
CD33+ HLA-DR -/low
1.44%
HLA-DR BV421
CD33 APC
CD33+ HLA-DRneg-lo gated
M-MDSCs
60.9 %
CD15 FITC
CD14 PerCP Cy5.5

Suppl. Fig. 4

Suppl. Table 1. Demographic table of the MS patients enrolled for the PMBC follow up study

**Epidemiological data**	**All MS patients n = 47**	**MS ≤30 days n = 30**	**MS >30 days n = 17**	**HC n = 26**
**Age**[a]	36.86 (9.03)	35.13 (9.44)	39.11 (8.12)	34.77 (10.28)
**Male/female (%female)**	15/32 (68.09)	10/20 (66.66)	5/12 (70.59)	12/14 (53.84)
**EDSS baseline**[b]	3 (2-4)	3 (3-4)	1.75 (1-2)	NA
**EDSS 12m**[b]	1 (0-2)	0.5 (0-1)	1 (0-2)	NA
**Gd+ lesions**				

HC: Healthy controls. [a] = mean ± SD. [b] = median ± interquartile range. NA: Not applicable.

Suppl.Table 2 Clinical and demographic data of MS followed up patients

Patient ID	Age	Sex	Time since relapse (days)	Treatment during follow-up	EDSS baseline	EDSS 12 months	MDSCs/MNCs baseline	MDSCs/MNCs 12 months
**I**	44	F	10	---	2	0	2.67	1.62
**II**	23	M	8	R	2	0	2.27	0.92
**III**	51	F	22	DF	4	1	0.52	0.87
**IV**	24	F	6	Cpx	3	0	2.00	3.47
**V**	30	M	5	Cc/DF	3	0	0.82	1.69
**VI**	50	F	21	Cc/DF	3.5	3	1.41	4.05
**VII**	32	F	33	Cc/Hf	4	0	2.32	3.14
**VIII**	42	F	10	Cc/Hf/Cpx	5	4	0.7	0.28
**IX**	40	F	32	Cc/DF	4	3	0.07	7.34
**X**	44	F	19	Cc/Cdb	4	0	0.72	0.89
**XI**	30	F	12	Cc/DF	4	1	0.36	0.88
**XII**	42	M	21	Cc	3	2	0.74	2.79
**XIII**	39	F	6	Hf	4	1	1.38	1.48
**XIV**	23	M	11	Cc/DF	4	0	0.15	1.11
**XV**	57	F	16	Cc	3	0	0.76	1.59
**XVI**	37	M	17	Df	3	0	1.41	7.71
**XVII**	29	M	13	Cc/DF/Hf	3	0	2.46	0.31
**XVIII**	37	M	14	Cc/DF	3	1	0.06	2.54
**XIX**	32	M	16	Cc/DF	3	0	1.37	5.26
**XX**	30	F	30	Cc/PG	3	1	1.11	1.44
**XXI**	36	F	12	Cc/DF	2	0	1.85	6.53
**XXII**	16	F	5	Ntz	3	2	0.30	1.09
**XXIII**	24	M	9	DF	2	0	3.39	0.95
**XXIV**	36	F	9	Ntz	4	1	3.44	6.16
**XXV**	39	F	8	If	5	1	1.7	1.27
**XXVI**	35	F	6	Atz	1	0	3.7	8.22
**XXVII**	43	F	7	---	2	1	3.82	2.30
**XXVIII**	35	F	14	Tf	5	1.5	0.14	4.73
**XXIX**	21	M	9	Tf	3	0	0.67	0.71
**XXX**	34	F	6	Cc/DF	3	3.5	1.02	0.97

Abbreviations: Atz: Alemtuzumab Cc: corticosteroids; Cdb: Cladribina; Cpx: Copaxone; DF: Dimethyl Fumarate; Hf: Hidroferol; If: Interferon; R: Rebif 44; Ntz: Natalizumab; PG: Plegridy; Tf: Teriflunomide.
