# Supplementary material for: Central and peripheral myeloid-derived suppressor cell-like cells are closely related to the clinical severity of multiple sclerosis
Source: Acta Neuropathol. 2023 May 27;146(2):263–82. doi: 10.1007/s00401-023-02593-x (PMC10329064; doi:10.1007/s00401-023-02593-x)
Supplement: Supplementary file 2 — Supplementary file2 (DOCX 16 KB) [file 401_2023_2593_MOESM2_ESM.docx]

**Supplementary figure 1. a:** Scheme of the HLA-DR cell diversity in MS lesions according to their fluorescence intensity. **b**: Representative quantification of HLA-DR intensity in a MS patient in order to identify the HLA-DR^low^ cell population based on the cut-off value.

**Supplementary figure 2. a:** Schematic representation of immunosupression assay with MOG-stimulated splenocytes and circulating Ly-6C^hi^ cell obtained from a different EAE mouse at onset. **b**: Immunosuppression assay using Ly-6C^hi^ cell-depleted splenocytes in co-culture with peripheral Ly-6C^hi^ cells isolated from a different EAE mouse at onset. **c**: After removing endogenous Ly-6C^hi^ cells from splenocytes, Ly-6C^hi^ cells obtained from the blood of EAE mice at onset were able to suppress the proliferation of T cells. **d**: Representative flow cytometry plots showing the immunosuppressive effect of circulating Ly-6C^hi^ cells over different cell population. Data from c-d are representative of five independent experiments, N = 10 mice.

**Supplementary figure 3**. **a**: Representative flow cytometry plots of circulating Ly-6C^hi^ cells at the onset of the clinical course from male EAE mice with differente SI. **b**: The abundance of Ly-6C^hi^ cells relative to the myeloid component in the peripjheral blood at the onset of the clinical symptoms in male EAE mice was inversely correlated with the SI. **c**: Representative flow cytometry plots of circulating Ly-6C^hi^ cells obtained at the peak of the symptoms from male EAE mice with differente recovery. **d**: In male EAE mice, the level of peripheral Ly-6C^hi^-cells at the peak of the disease was directly correlated with the percentage of symptom recovery. N= 23 in c; N = 21 in d.

**Supplementary figure 4**. **Gating strategy for human M-MDSCs analysis**. After aggregates exclusion (*singlets*gate), Zombie-NIR negative events were gated as *Live* cells, which include counting beads too. Live Mononuclear cells were then gated (*MNCs* gate) excluding cellular debris and counting beads. Next, monocytes were selected for subsequent analysis on an FSC vs SSC dot-plot. HLA-DR and CD33 expression was analyzed on monocytes and HLA-DR ^-/low^ CD33^+^ cells were gated in order to analyze CD14 and CD15 expression. M-MDSCs were finally identified as HLA-DR^-/low^ CD33^+^ CD14^+^CD15^-^cells.
